# Assessing the reproducibility of a bioimage analysis workflow characterising tissue flow in *Drosophila*

**DOI:** 10.1111/jmi.70125

**Published:** 2026-06-18

**Authors:** Mélodie Ambroset, Cesar Augusto Valades‐Cruz, Libert Brice Tonfack, Rocco D'Antuono, Caterina Fuster‐Barceló

**Affiliations:** ^1^ CHU Nantes, CNRS, INSERM, BioCore, US16, SFR Bonamy Nantes Université Nantes France; ^2^ UR BIA, INRAE Nantes France; ^3^ PROBE Research Infrastructure BIBS Facility INRAE Nantes France; ^4^ Institute of Hydrobiology Chinese Academy of Sciences Wuhan China; ^5^ Unit of Plant Physiology and Improvement Laboratory of Biotechnology and Environment Department of Plant Biology Faculty of Science University of Yaounde 1 Yaounde Cameroon; ^6^ Crick Advanced Light Microscopy STP The Francis Crick Institute London UK; ^7^ Department of Biomedical Engineering School of Biological Sciences University of Reading Reading UK; ^8^ Department of Molecular Life Science Universität Zürich Zürich Switzerland

**Keywords:** bioimage analysis, data analysis, deep learning, methods, microscopy, physical modelling, reproducibility

## Abstract

Reproducibility challenges in bioimage analysis are common. The proliferation of tools and workflows creates a complex ecosystem that is difficult to reproduce without deliberate, explicit reporting and support.

We assess the reproducibility of Gehrels et al.(2023) as a representative bioimage analysis workflow. We do not evaluate the biological claims; instead, we examine the reproducibility of the bioimage analysis methods used and the implications for current peer‐review practices in the field. Although some data were deposited in the BioImage Archive and code was available on GitHub, the materials were insufficient to completely reproduce the analysis. Moreover, the repository lacked the configuration to enable straightforward replication of the workflow or results.

This case study highlighted how incomplete reporting can significantly hinder the reproduction of bioimage analysis workflows. Because scientific progress relies on verifiable replications, insufficiently reproducible results can erode confidence in the scientific record.

## 1 INTRODUCTION

Reproducibility in microscopy is still a current problem for the scientific community, and concerns have been raised regarding common mistakes such as incomplete metadata reporting, insufficient validation of instruments, inadequate documentation of sample preparation, and poorly described detection and acquisition settings, among other issues.^1^ In addition to this, transparency in image processing also needs to be addressed, including the full description of all processing steps, the archiving of raw data, and the preservation of the metadata and image formats.^2^

A closely related challenge is the reproducibility of image analysis workflows. Mishandling of image data can lead to misleading results that weaken scientific conclusions. Common errors or mishandling include, for example, overusing a single fluorescence channel for both segmentation and measurement, inappropriate contrast enhancement or cropping, and incorrect bit‐depth conversion, all of which can distort quantitative outcomes.^3^ To address these issues, reproducible analysis requires that workflows be documented and shared in detail, ideally including: (i) the workflow code, (ii) a clear description of each processing step and software version, and (iii) access to the original image data.^3^

However, common obstacles are the lack of formal training,^4^ lack of awareness among researchers regarding how image processing steps affect data integrity,^5^ and the lack of incentives to develop well‐documented code and maintain open‐source software.^6^ This lack of accountability is, in part, a result of missing editorial policies and insufficiently rigorous peer review, which often fail to enforce detailed reporting standards.^5^ Strengthening both author proficiency through training and editorial oversight during the peer‐review process is therefore essential to foster reproducibility in microscopy and image analysis.

During the annual bioimage analysis workshop organised by the Global Bioimage Analysts’ Society (GloBIAS) in 2024,^7^ participants joined a reproducibility workgroup that aimed to analyse and reproduce part of scientific publications proposed by GloBIAS members and the session chair, in order to assess the reproducibility of different bioimage analysis workflows, using the descriptions and code provided in the original article. The session chair selected articles from major journals that presented interesting bioimage analysis methods and assigned one to each group of participants to critically analyse the reproducibility aspects of the publications.

This work presents a qualitative report on the reproducibility of the bioimage analysis workflow associated with a publication, titled ‘Curvature gradient drives polarised tissue flow in the *Drosophila* embryo’,^8^ which described the onset of polarised tissue flow in *Drosophila*, and identified how both local curvature and actomyosin contractility determine the direction of the flow.

The authors of the analysed publication^8^ used *Drosophila* embryos to study the physical mechanisms of tissue flow and the events, such as accumulation of myosin and changes induced by genetic perturbations, which influence the initiation of asymmetric movements in the epithelial cell layer at an early stage. The cited literature indicates that, in other organisms or development stages, the polarised tissue flow is due to the presence of specific groups of cells that constrain the movement of remaining cells or to the presence of fixed points that generate forces, driving the direction of tissue movements along the surface of the embryo or causing its invagination; in this context, the authors of the original manuscript tried to identify the specific mechanisms acting in *Drosophila* embryo. In particular, their main goal was to determine whether the presence of forces generated at the posterior pole at an earlier stage initiate, or are at least necessary, for the generation of polarised tissue flow. In the original study, the authors used microscopy experiments on several genetic variants to measure the geometry of the embryo, analyse tissue velocity and myosin distribution, and compared the results with simulated models.

To evaluate these findings from a computational perspective, we mapped the authors’ methodology into a structured bioimage analysis pipeline. Figure 1 illustrates this workflow, beginning with the microscopy modalities used for data acquisition (Figure 1A), followed by segmentation (Figure 1B) and tissue flow quantification (Figure 1C) and ending with the extraction of biophysical parameters (Figure 1D). It should be noted that while the original study includes biological modelling, our representation and subsequent analysis focus specifically on the image processing and data extraction components of the pipeline, as these constitute the primary technical scope of this manuscript.

**FIGURE 1:**
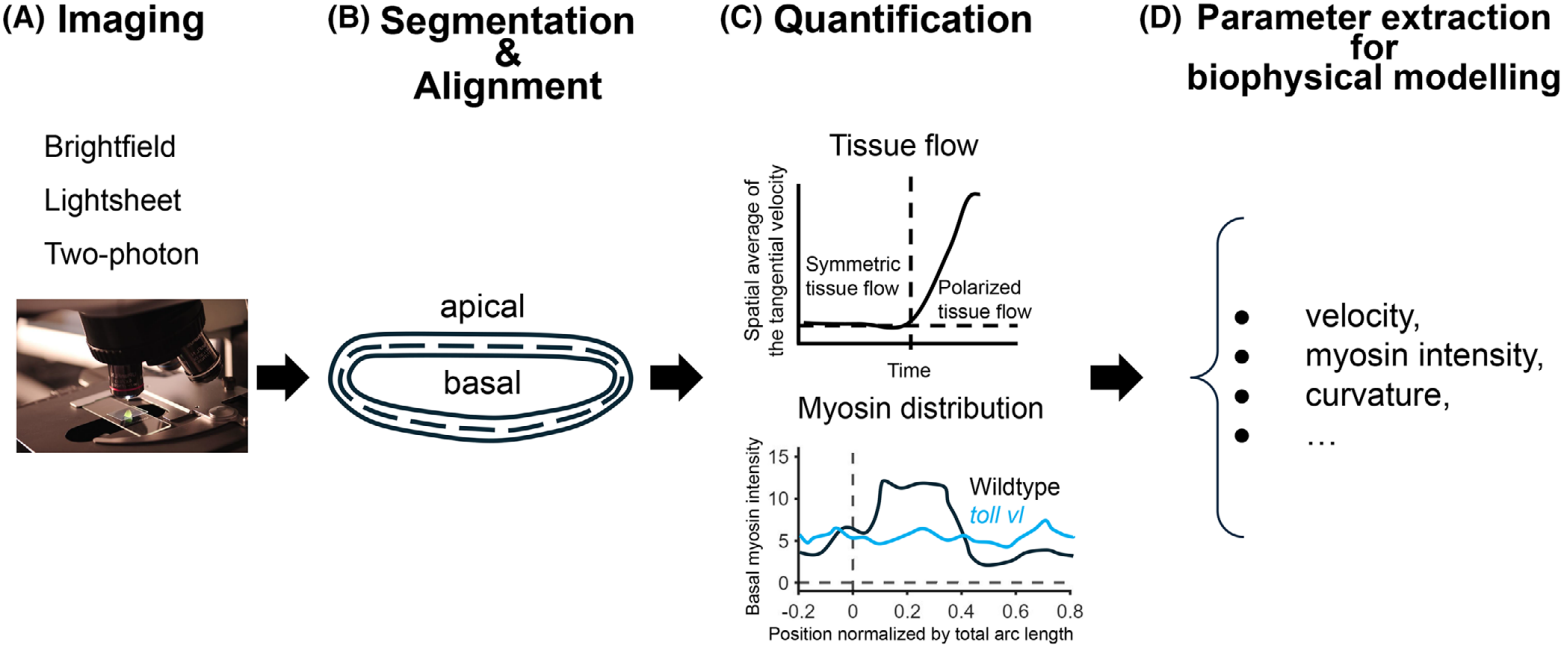
Overall bioimage analysis workflow of the analysed publication. (A) Imaging of embryos using brightfield, lightsheet, and two‐photon microscopy (Creative Commons CC0); (B) segmentation and alignment of the epithelial contour along the apical–basal axis; (C) quantification of tissue flow and myosin intensity distribution along the normalised arc length (representative sketches inspired by the original publication, not real data); (D) parameter extraction for biophysical modelling.

We emphasise that this work focuses solely on the reproducibility of the analysis workflow and does not question the biological conclusions, which were already validated by experts during the peer‐review process of the original article.

Our workgroup dissected the original publication and all other available materials with the aim of identifying the steps of the bioimage analysis workflow proposed by the authors. Despite the availability of some data sets and code, the reproducibility of the complete image analysis workflow was not possible for us, due to the lack of full documentation and original data used to create the figures in the publication. Since the purpose of this current work was to describe the level of reproducibility in scientific publications, we focused on classifying the content of the original article, identifying strengths and weaknesses, according to a community‐developed checklist for the publication of image analysis workflows.^9^

## 2 MATERIALS AND METHODS USED IN THE ORIGINAL PUBLICATION

In this section, we present our understanding of the materials and methods used in the original publication, with a focus on reconstructing the bioimage analysis workflow step by step. To assess the reproducibility of each component, we carefully reviewed not only the main manuscript but also the supplementary materials and the publicly available source code provided by the original authors (https://github.com/HiBandan/modelTissueFlow).

While the article outlines the general structure of the analysis, several methodological details were either implicit or not fully documented. As a result, reconstructing the workflow required a detailed examination of code functions and of the implementation logic in the GitHub repository. In some instances, we had to infer specific procedures based on the structure and behaviour of the code. We acknowledge that our interpretations may not fully capture the original intentions of the authors; however, every effort was made to interpret the provided resources faithfully. This reconstruction forms the basis for our evaluation of the reproducibility and transparency of the image analysis pipeline.

### 2.1 Data acquisition and availability

#### 2.1.1 Microscopy and image acquisition methods in the original publication

Three microscopy modalities were used in the original publication: brightfield, lightsheet, and multiphoton microscopy. Brightfield movies (one image per minute for 2 to 3 h) were acquired using a Zeiss Axiovert 200 M inverted microscope equipped with a 20×/0.75 NA objective. The genetically encoded fluorescent tags mScarlet for plasma membrane and GFP for myosin were imaged with lightsheet and two‐photon microscopy. Lightsheet imaging was performed using a Zeiss Lightsheet Z.1 microscope with a 20×/1.0 NA W UV‐Vis detection objective and two 10×/0.2 NA illumination objectives. Time‐lapse volumes (105 µm depth; 1.5 µm z‐steps; one volume collected every 30 s) were acquired. Two‐photon movies (single plane, one frame every 30 seconds) were acquired using a Nikon A1R MP+ multiphoton microscope equipped with a 25×/1.10 NA water‐immersion objective.

#### 2.1.2 Image data availability in the original publication

Some example data was provided through the EBI BioImage Archive under the ID S‐BIAD602,^10^ for which we show the directory tree in Figure S1.

After careful analysis of the deposition, two imaging modalities appear available: lightsheet microscopy and two‐photon microscopy. The dataset is organised in a structured repository by imaging modality (lightsheet, two‐photon), genotype (wild‐type, *toll vl*), and embryo identifiers.

For the lightsheet modality, one multi‐dimensional TIFF file (.tif) is provided for one wild‐type embryo. The filename seems to encode the acquisition date, the two fluorescent tags, the experimental condition, temperature, spatial resolution, channel information and time point. The file is a volume (z‐stack of 72 slices) cropped around the embryo, for a single time‐point (frame 10) and a single channel (myosin).

For the two‐photon modality, data are available for both one wild‐type and one mutant embryos. For the wild‐type embryo, two .tif time‐series (78 time points) are available: one for the membrane (MEM) and one for the myosin (MYO) channel. The file names contain partial experimental details, such as acquisition date, fluorescent tags, immersion medium and channel. In an additional ‘MARKERS’ subfolder, an image of a single myosin frame (21/78, F_020.tif) is provided, where pixels not belonging to the epithelium (such as those of the vitelline membrane, pole cells or inside of the embryo) have been masked (set to zero). For this embryo only, an additional RTF file containing basic acquisition information (laser powers, objective magnification, pixel size, acquisition speed) is included. For the *toll vl* embryo, only an image of a single time point (1/95, F_000.tif) is provided, for the membrane channel exclusively.

Additionally, two two‐photon movies were included in the GitHub repository (https://github.com/HiBandan/modelTissueFlow/tree/main/examples/movies). Both movies are organised in ‘MEM’, ‘MYO’ and ‘MARKERS’ folders (respectively for membrane, myosin, and masked myosin channels), each containing individual TIFF files for a few consecutive frames (e.g. F_044.tif). Movie 1 frames are from the same wild‐type embryo present in the S‐BIAD602 archive, and Movie 2 frames are from another original file, ‘210128_GAP43mSc_sqhGFP_34_3.nd2’ (based on metadata), which is not part of the S‐BIAD602 dataset.

### 2.2 Code availability

The authors also made their analysis code available through a public GitHub repository (https://github.com/HiBandan/modelTissueFlow) under a GPL‐3.0 license. The code is structured in modules, object classes, and functions. This effort towards open code sharing is commendable and aligns with best practices in computational biology.

The repository includes a .txt file listing the requirements for environment setup, which is a helpful inclusion. However, the file does not specify package versions.

The software is also distributed on PyPI as ‘modelTissueFlow’ and can be installed via ‘pip install modelTissueFlow’. The PyPI release shows versions up to 3.1 (29 March 2023) with earlier versions dating back to August 2022. This means that the installation can be done either via PyPI or from source code by downloading the repository.

In regard to the documentation and how to use this code, the README file explicitly includes a short statement of scope, a pointer to example scripts and a contact email for issues. However, the exact runtime environment specifications (OS, Python version, CPU/GPU assumptions) are not described or presented in a documented ‘quickstart’ command to reproduce a specific figure or result from the manuscript.

Despite there being a link to the paper dataset, the repository landing page focuses on installation and general description and there is no link to which data has been used in the code or any linkage to connect the code to the publication's dataset.

### 2.3 Image analysis workflow

#### 2.3.1 Image segmentation of the epithelial layer

In the original study, the workflow includes the segmentation of epithelial tissue (Figure 2). This was a key preprocessing step for quantifying both myosin localisation and tissue flow dynamics in *Drosophila* embryos during early morphogenesis. Two‐photon sagittal images from membrane and myosin channels were used to extract the apical and basal contours of the epithelial sheet (Figure 2A).

**FIGURE 2:**
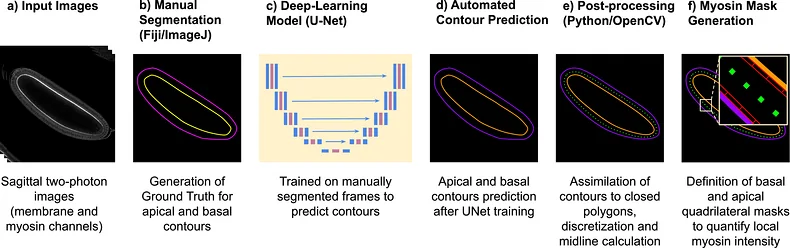
Workflow for epithelial segmentation and myosin quantification from sagittal two‐photon images of *Drosophila* embryos. The segmentation pipeline includes (A) input images from membrane and myosin channels;^8^ (B) manual segmentation of apical (magenta) and basal (yellow) contours in selected frames to generate ground truth; (C) training a U‐Net model (for which detailed architectural and training parameters were not reported) for automated segmentation of the contours; (D) automated prediction on full time‐lapse series of the apical (purple) and basal (orange) contours; (E) post‐processing resulting in the midline calculation (green) of the discretised closed outlines (purple and orange); and (F) construction of the quadrilateral masks (red) to later quantify local myosin intensity at basal and apical nodes.

To obtain accurate segmentations,the authors began by manually annotating a subset of image frames using ImageJ/Fiji.^11^ The contours of both the apical (facing the exterior) and basal (facing the interior) epithelial surfaces were traced manually in selected frames of the time‐lapse movies (Figure 2B). These annotated contours were then used as training data to develop a deep learning segmentation model based on a U‐Net architecture^1^ (Figure 2C).

Once new images were segmented with the specified deep learning model (Figure 2D), the predicted apical and basal contours were post‐processed to be assimilated to closed polygons (Figure 2E). The two closed outlines were each discretised into 100 evenly spaced nodes such that each basal node had a corresponding apical node. From each pair of nodes, a midline node was calculated at the average position (green dot in Figure 2E). For each apical and each basal node, a quadrilateral region was defined, with a fixed height (approximately ten pixels, corresponding to the height of the myosin signal), and a width determined by the distance between neighbouring nodes (Figure 2F). While the midline itself was not used to construct intensity masks, it served as a geometric reference for aligning and indexing the node pairs. These quadrilateral regions were used as masks for quantifying local myosin intensity along the epithelial sheet.

Further processing steps, such as alignment of embryos via ellipse fitting on the apical surface and conversion to arc‐length coordinates, were performed to standardise analysis across embryos and time points.

#### 2.3.2 Characterisation of tissue flow (speed and spatial profile)

The authors next sought to characterise tissue flow on the sagittal two‐photon images with two different methods.

The authors first tracked the position of the pole cells as a proxy for quantifying the motion speed of the posterior tissue. Pole cell tracking was done with the ImageJ/Fiji software.^11^ On the membrane fluorescent channel of the images, they manually tracked the position of a central pole‐cell, starting at the time when the cellularisation front passes the nuclei in the dorsal posterior part of the embryo. When the tissue invaginated, the position of the pole cell was extrapolated towards the vitelline membrane to only account for tangential movement.

To extract the spatial profile of the flow, they next performed a Particle Image Velocimetry (PIV) analysis on pairs of consecutive frames using the *openpiv* Python library. They measured the velocity of the flow tangential to the midline of the epithelium, considering that its arc‐length increases in the clockwise direction and normalising it to the total length of the midline. The ‘zero’ reference of the arc‐length coordinate is located at the posterior pole, where the principal axis of the ellipse fitted on the embryo outline (Section 2.3.1) intersects with the midline. They then spatially averaged the velocity of the flow to a single value per time point to derive its polarity (symmetrical when the average is zero and there is as much clockwise (positive) as counterclockwise (negative) flow, polarised otherwise).

#### 2.3.3 Myosin quantification

As the last step of the workflow, the authors sought to quantify the apical and basal distribution of myosin in the embryos to assess its impact on tissue flow.

For the two‐photon sagittal images, using a Python script, the authors averaged the pixel intensities of the myosin channel in each quadrilateral mask denoted region covering either apical or basal myosin (see Section 2.3.1).

They also sought to compare apical myosin expression in wild‐type and one of the genetic variant (*fat2*) embryos to make sure that the lower tissue speed observed for the latter was not due to lower myosin expression. Given that *fat2* embryos were too round to align and consistently capture apical myosin domains with two‐photon microscopy, they used lightsheet microscopy to image it in the posterior part of embryos. For these lightsheet images, using ImageJ/FIJI, they first resliced the z‐stack containing slices in the posterior‐anterior orientation perpendicularly to the the dorsoventral axis. To locate the axis, they used the direction of the pole cells movement during the fast phase and the location of the mesoderm invagination. On the resliced stack, they identified the onset of polarised flow as the time when a fast movement of the pole cells first occurs. For each of three selected time points at 2, 2.5 and 3 min after the onset of polarised flow, they traced a freehand line spanning the punctate myosin expression to measure the length of the apical myosin domain and average myosin expression. The measurement was performed three times for each of the three selected time points and the nine measurements were averaged to mitigate the effect of noise. The average myosin intensity was then normalised by the average basal myosin intensity (measured 5 min after cellularisation, well before the first appearance of apical myosin puncta).

#### 2.3.4 Parameter extraction for biophysical modelling of embryo tissue flow

The analysed publication includes very detailed physical modelling to explain the behaviour of tissue flow, based on the effect of applied forces on tissue velocity. The model, derived in the supplementary materials of the original article, describes the movements of epithelial tissue with equations including terms to account for tissue viscosity, friction with the vitelline membrane, active tension based on myosin content, active moment generated by differences of myosin content at the apical and basal sides of the embryo epithelium, and tissue curvature imposed by the eggshell shape. The parameters of the equations were fitted using experimental data acquired with two‐photon microscopy, averaged over all the embryos for the time points of interest.

To extract model inputs, the authors used their custom Python script to create a midline contour composed of a set of midline nodes located midway between each pair of respective apical and basal nodes. At these midline nodes, several parameters were extracted: the tissue velocity tangential to the midline as computed with PIV (Section 2.3.2), the total myosin intensity (sum of myosin intensity at apical and basal nodes, Section 2.3.3), the active moment (corresponds to the difference between myosin intensity at apical and basal nodes multiplied by the distance between the midline node and either apical or basal node), and the curvature (spatial derivative of the angle between the pairs of adjacent midline segments).

The development of numerical simulations would have been beyond the scope of this work, which focuses on the image analysis workflow. Thus, we do not discuss modelling any further.

## 3 RESULTS

### 3.1 Analysis of image data in the original manuscript

In the methods of the main text, the laser wavelengths used for lightsheet and two‐photon imaging were provided, but the laser powers were not indicated. The laser brand was mentioned for the two‐photon imaging, but not for the lightsheet system. In addition, although there is mention of the used objective lenses in the original publication, it would be useful for reproducibility to also include the brand and model of each objective, as well as to specify in all the cases whether they are air, oil, or water immersion. Including this information is important because it allows readers to identify whether the experimental conditions and acquisition settings were consistent for a quantitative analysis of microscopy data.^12^ Indeed, the optical corrections of each lens (e.g., achromatic, apochromatic, etc.), its working distance, its transmittance for different wavelengths, and other optical properties influence the image acquisition and therefore the possible analysis.^2^

Some of the imaging data from the original study are publicly available in the GitHub repository and through the BioImage Archive under accession ID S‐BIAD602,^10^ as described in Section 2.1.2. Publishing data on such an archive represents a significant strength as it can allow data reuse.

However, while the original article states that ‘the raw image files’ and ‘the datasets generated and analysed during the current study are available at the S‐BIAD602 BioImage Archive’,^8^ (p. 10), a closer examination reveals that it does not include all the images and metadata used to produce the figures, but contains only a small subset of the original data.

The lightsheet data provided only includes a volume for a single time point and a single channel (myosin) for a wild‐type embryo, and this file does not contain metadata. It is understandable, given the large size of the data produced with lightsheet microscopy, that only part of the dataset was deposited. Nevertheless, the available data are limited, considering that movies of at least 20 min have been acquired, for at least two channels, and that 5–7 embryos per condition were used in the publication.

The two‐photon data is provided in two different forms: images of single consecutive time points on GitHub and single‐channel full time‐series on the archive. For the *toll vl* embryo, a single slice of the membrane channel is provided. Both membrane and myosin channels are provided for the wild‐type embryo, but the metadata indicates that three channels were originally acquired. This, as well as the mention of a ‘.nd2’ original image in the metadata (see Section 2.1.2), seems to indicate that the data provided is not raw and has at least been split and converted.

Another advantage of the BioImage Archive repository is that it requires a description of the data and microscopy settings. In this dataset, such information was included for the two‐photon microscopy, but it was missing for the lightsheet data. An RTF file containing some experimental information was also provided for the two‐photon dataset. Overall, the RTF summarises only a minimal amount of acquisition metadata (laser powers, objective magnification, pixel size, acquisition speed), presented as unstructured notes. One commendable effort from the authors is that some of the filenames include experimental information. However, since no file naming convention is provided, it is up to the reader to decipher what each piece of the filename refers to by crossing it with plausible explanations gleaned in the text or in other documents such as the RTF file.

The brightfield microscopy modality is absent from the archive and GitHub repository. The Material and Methods section of the original manuscript simply mentioned that it was used to characterise the direction of tissue flow for wild‐type and *toll vl* embryos. The supplementary figure about this experiment however revealed that Differential Inference Contrast (DIC) microscopy was used. While this distinction was only specified in the figure legend, we think that including it in the main text or methods section would have significantly clarified the method used. No DIC example image is explicitly mentioned in the article nor provided as supplementary data, and we found no explanation on how tissue direction was determined on DIC movies. We thus did not analyse this supplementary experiment any further.

Furthermore, not all data required to fully reproduce the analysis pipeline are present. For example, key components necessary for validating the image segmentation process are absent, such as the manually annotated ground truth images used for training the U‐Net model.

Other types of data, such as the trained network weights, or the resulting predicted segmentation masks, are not included either in BioImage Archive or in similar repositories to store weights and DL models, like the BioImage Model Zoo^13^ or HuggingFace (https://huggingface.co/). This limits the ability to benchmark model performance or evaluate downstream processing results. These limitations are further explored in the segmentation section below (Section 3.3.1).

In conclusion, the data provided only represents a minimal part of the whole data used in the original publication. Therefore, reproducing the original workflow either in part or end‐to‐end proved difficult given the limited data availability. This limitation will be analysed in more depth by describing the specific parts of the workflow.

### 3.2 Code reproducibility and analysis

Over the course of this work, we attempted to install and run the code on multiple systems with different operating systems and Python configurations and environments (see Supplementary Material S.2). Across macOS (Apple Silicon) and Windows, both installation routes described by the authors (via PyPI and source installation from Git clone) failed to yield a runnable, importable environment. In particular, the PyPI installation path failed during metadata generation while the source installation path progressed further but exposed a series of import‐time failures caused by missing or undeclared runtime dependencies. In all cases, dependency conflicts or deprecated packages prevented the successful execution of the pipeline. Additionally, the repository has not been updated in over 3 years, and usage instructions or examples to guide users through the workflow are minimal. Taken together, the issues we encountered suggest that the repository does not currently provide a fully specified, version‐pinned environment that can be recreated directly from the provided files. A detailed system‐by‐system record of commands, error messages, and attempted fixes is provided in Supplementary Material S.2 and Table S1.

Together, these limitations impacted our ability to evaluate the pipeline in its original form and highlight the importance of maintaining runnable environments and versioned dependencies for long‐term reproducibility. More broadly, our experience illustrates that providing public code alone is often insufficient for computational reproducibility unless the authors also supply an executable ‘reference’ environment, as well as a minimal end‐to‐end example that allows users to validate that the installation reproduces at least one expected intermediate output.

### 3.3 Reproducibility of the image analysis workflow

#### 3.3.1 Reproducibility of the epithelial layer segmentation

Despite the central role of segmentation in the analysis pipeline, we encountered significant challenges in reproducing this step, based on the information provided in the original publication and supplementary materials. The manual annotations used as ground truth for training the segmentation model were not shared, and no guidance was given on the precise annotation protocol. Consequently, even though a small subset of the raw two‐photon image data is available, reproducing the training dataset from scratch would require making subjective decisions about where to draw apical and basal contours, potentially introducing inconsistencies and bias.

Furthermore, the U‐Net model used for segmentation is not provided in a trained form, nor are the specific details required to reimplement or retrain it. The original publication does not report which images were used for training versus testing, the exact network architecture (beyond stating that it was based on a U‐Net), or the hyperparameters used (e.g., learning rate, batch size, number of epochs, optimizer). This absence of key documentation makes it infeasible to replicate the segmentation model, especially for researchers without prior expertise in deep learning for bioimage analysis.

The post‐processing steps that convert the predicted apical and basal contours into closed polygons and create the quadrilateral masks for myosin quantification are only briefly described in the original text, making them difficult to interpret. With no straightforward way to test the code, we inferred the expected characteristics of the contours and masks through manual inspection of the relevant functions (see Section 3.3.3). No figure illustrating the post‐processing details was available in the article that would have made it easier to orient our search or confirm our assumptions. In the same way, we located the function that seems to fit an ellipse on the apical polygon to align the embryo in the *inOutTools.py* module of *modelTissueFlow* but could not run the code to check the results on the available two‐photon images. Overall, the lack of clear and complete implementation details limited our ability to reconstruct the full segmentation workflow.

#### 3.3.2 Reproducibility of the characterisation of tissue flow

Our intention was to perform both tissue flow speed and tangential velocity measurements on the data provided by the authors on Bioimage Archive (Section 2.1), by using the provided code on the GitHub repository. As explained in Section 3.2, we failed to install a working conda environment due to dependency issues that were impossible to resolve since Python and library versions were missing. PyPI and building from source also failed, despite attempts to down‐version libraries or change outdated library names. The supplementary movie S3 of the original article seems to indicate that pole‐cell tracking was done on the membrane channel of the two‐photon movies. Despite there being one two‐photon movie with both myosin and membrane channels on the archive, and folders containing individual frames for two different movies in the GitHub repository, there is no available track files or other type of positions lists to allow the re‐plotting of the cell movement. Moreover, there is no clear indication on how to select a ‘central’ pole‐cell for tracking, nor on how to precisely track it when the pole cell moves out of the plane or when the tissue invaginates. We assume that a general ‘midpoint’ location of the pole‐cell pool is used, but cannot confirm it without coordinates lists. The authors also give no indications on how such coordinates are recorded, making it difficult to reproduce this manual tracking method in ImageJ/FIJI.

The PIV analysis is described extremely briefly in the publication, the authors solely state that they used the *openpiv* Python library to perform PIV on subsequent frames and extract the tissue velocity tangential to the midline of the epithelium. We assume that PIV was performed on two‐photon membrane channels, and that it measures tissue‐level texture flow rather than actual tracer particles. We searched the source code (specifically *analysisModule.py*) for PIV related functions. Three functions seem to calculate velocity vector fields for frame pairs and interpolate them around boundary (apical, basal, midline) nodes, before decomposing them into tangential or normal motion relative to the direction of the boundary. There is no explanation in the text on how parameters for these functions are set, such as the size and overlap of interrogation windows, the size of the search area, or filtering methods for signal validation. While reproduction of this analysis would have been possible with a working code, we believe that a more extensive documentation in the text, the supplementary material or the code would also have been beneficial to understanding the steps and parameters involved.

#### 3.3.3 Reproducibility of the myosin quantification

To better understand the generation of masks in which myosin intensity was quantified on two‐photon images, we searched the source code and located functions in the modelTissueFlow analysis module that seem to construct quadrilateral regions at apical and basal nodes. These polygons—grouped into apical, semi‐apical, basal, and semi‐basal masks—likely serve as regions of interest for myosin intensity quantification (Figure 2F). While this code gave us (experienced bioimage analysts) hints about the geometric logic of mask generation, missing documentation, parameter values (e.g. layer widths), and pipeline integration made practical reproduction impossible. The code's minimal comments and absent documentation, combined with installation failures, made myosin quantification on two‐photon images impossible to reproduce.

For the particular case of myosin quantification on lightsheet microscopy images, only a single time point volume of the posterior area of a wild‐type embryo was available from the Bioimage Archive. This file has no metadata attached. From its name (‘221114_GAP43mSc_sqhGFP_raised18_T23_1_5um_2_myo_t10’), ‘t10’ most likely describes the time point, but it is impossible to deduce what it corresponds to (frame number of the full acquisition movie, time since cellularisation, time since first apical myosin apparition).

The stack is oriented along the posterior‐anterior axis. It is impossible to reslice it like the authors did without the full movie, since there is no way to deduce where the dorsoventral axis is from the direction of movement of the pole cells or the mesoderm invagination. Arbitrarily reslicing the stack from the left or from the top did not allow us to see apical myosin expression, but it is possible that it has not yet appeared at this time point. A hyperstack of relevant time frames (e.g. cellularisation, 5 min after cellularisation, first polarised movement, 2, 2.5 and 3 min after polarised movement), combined with a set of FIJI Regions Of Interest, or an illustration of the manually drawn lines both on the apical myosin domain and the basal myosin to which measurements were normalised to would have greatly improved the reproducibility of myosin measurement for lightsheet images.

### 3.4 Evaluation of the bioimage analysis workflow according to the community reproducibility checklists

Following Schmied et al.,^9^ we evaluated the original study by Gehrels et al.^8^ against the ‘Machine Learning (ML) Workflows’ and ‘New Workflows’ checklists.

For the ML Workflows checklist (Figure 3, top), the reproducibility level is minimal. This portion concerns the training of a U‐Net for apical and basal contour segmentation, a critical step in the pipeline, which is only detailed in the supplementary material of the analysed publication, not in the main text. In practice, none of the ML‐specific items are fulfilled in the main manuscript; the only element present is the name of the original U‐Net architecture, which is only mentioned in the supplementary material. There is no ‘Access to the model’, ‘Example or validation data’, ‘Training and testing data and metadata’, ‘Standardized model’ format, ‘Cloud hosted or container’, or commented, versioned code associated with this step.

**FIGURE 3:**
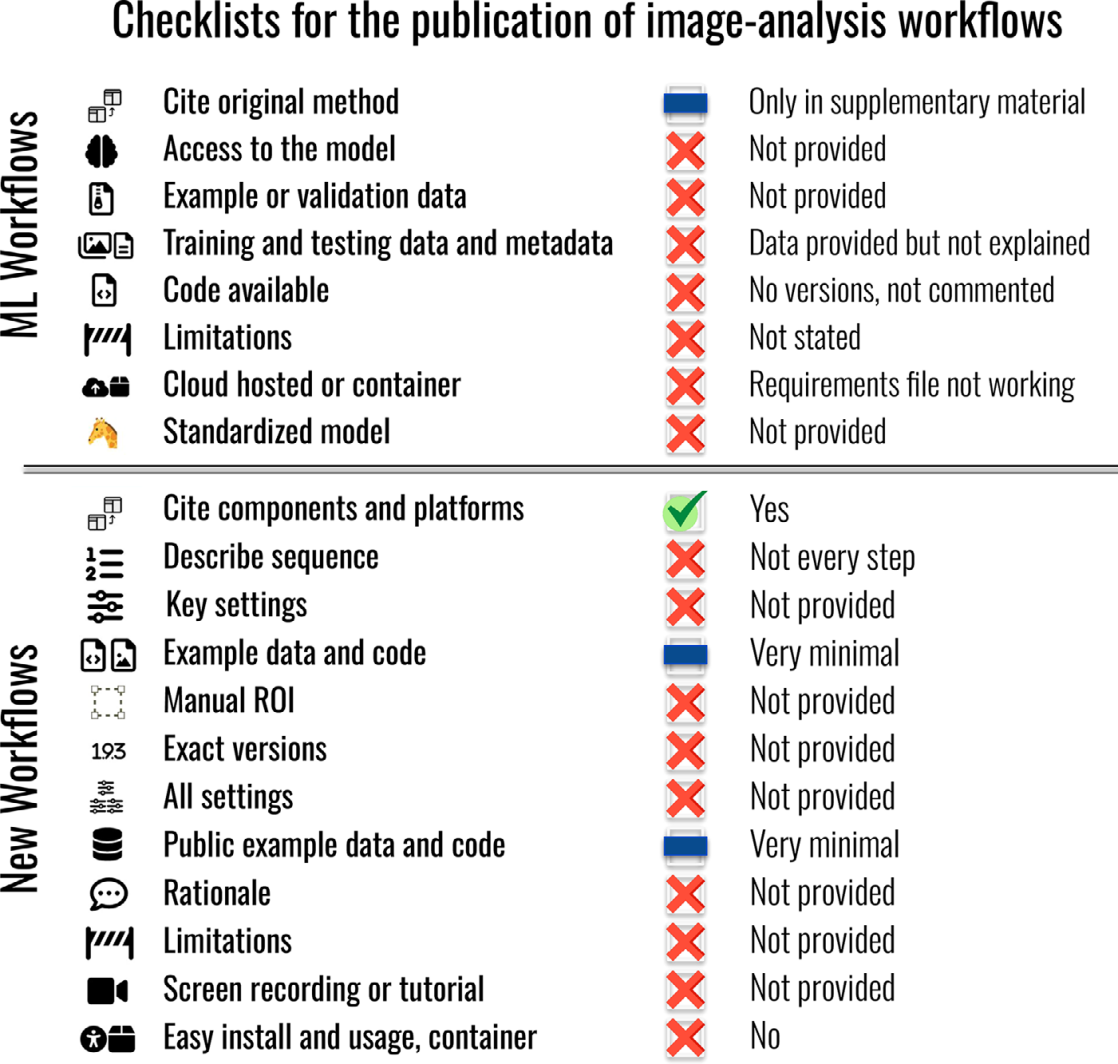
Evaluation of the replicability and reproducibility of the bioimage analysis workflow of the article ‘Curvature gradient drives polarised tissue flow in the *Drosophila* embryo’,^8^ based on the guidelines in ‘Community‐developed checklists for publishing images and image analyses’.^9^ Specific items of the analysed publications are scored as not provided (red cross), provided in a minimal or insufficient way (blue line), or provided (green tick). Important note: this analysis is an evaluation of the availability of documentation for the reproduction of an image analysis workflow, not an evaluation of the validity of the biological story described in the chosen publication. **Top**: scores according to the ‘ML Workflows’ checklist. **Bottom**: scores according to the ‘New Workflows’ checklist.

The complete end‐to‐end pipeline primarily falls under the ‘New Workflows’ checklist. As shown in Figure 3 (bottom), only one item is fully satisfied: ‘Cite components and platforms’. Two items are present but minimal: ‘Example data and code’ (as discussed in Sections 2.1 and 2.2) and ‘Public example data and code’ (Sections 2.1 and 2.2). All other items, such as ‘Exact versions’, ‘Key settings’, ‘Limitations’, ‘Rationale’, ‘Manual ROI’, and ‘Easy install and usage, containerization’, are not provided. Taken together, the workflow scores insufficiently across both checklists: a handful of items are present (often minimally), while most required elements for independent, executable reproduction are absent.

### 3.5 Citation follow‐on analysis

We sought to establish whether other publications had tried to reproduce the image analysis performed in Gehrels et al.^8^ or used the available code. To this end, we located the quotes in the 34 publications (as of submission date of this work) citing the article, of which most were research articles or preprints (Figure 4A). We attributed tags to publications depending on the nature of the citation. As shown in Figure 4B, the following publications tended to cite the theme (i.e. tissue flow in embryogenesis, 22.2%) or the general conclusion of the article (e.g. that the geometry or curvature of the embryo influences tissue flow, 75.0%), and one work (2.8%) cited a specific result of the original article, supporting their scientific hypothesis. Most often, the citation supported the general introduction or conclusion of the original article and treated the findings as established background knowledge (see ‘Quote’ and ‘Section’ columns of Table S2). No reference to specific (e.g. mention of the GitHub repository) or generic (e.g. segmentation, myosin quantification) methods used in the article were found and it seemed that no publication attempted reproducing any part of the article, lending no support to our reproduction efforts.

**FIGURE 4:**
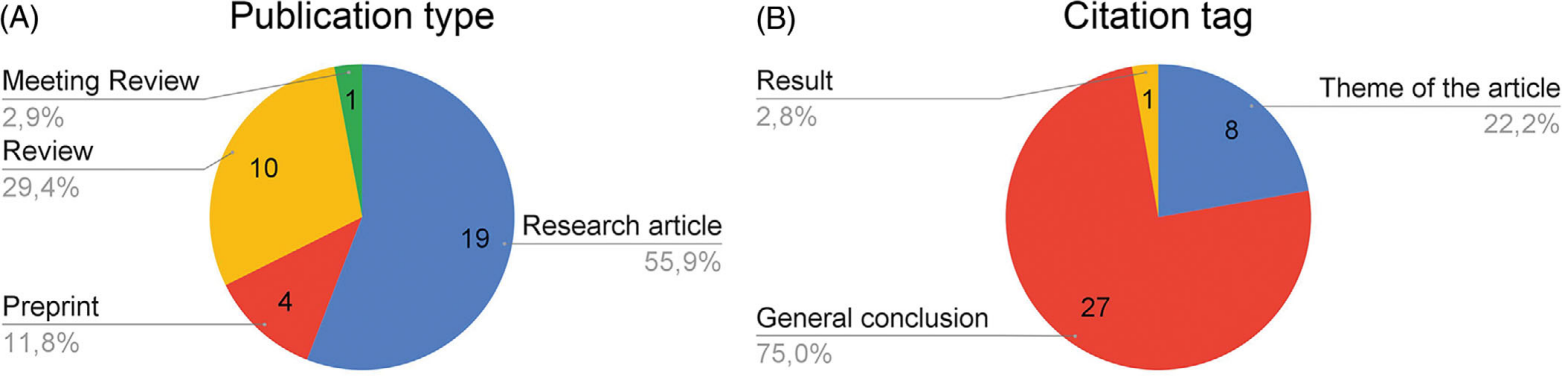
Citation analysis for the original publication. (A) Type of publications citing Gehrels et al.^8^ (B) Tags characterising the citations (one citing publication can refer to the same article multiple times).

To determine whether a biological research article that is neither a protocol nor a methods paper could be cited for its image analysis method, we examined the citing literature of a conceptually related study^14^ cited by Gehrels et al.,^8^ that uses similar methods—quantitative image analysis and biophysical modelling—to investigate the reorientation of planar cell polarity during *Drosophila* wing morphogenesis. While this work accumulates a total of 560 citations, we chose to consider only the 118 items published during the 3‐year period (2010‐2013) after its publication for a fair comparison. We then only explored conference, research and protocol articles, where the bioimage analysis method was most likely to have been mentioned, excluding reviews or commentaries. Out of the 67 analysed papers (Table S3), our search yielded 13 articles citing the image analysis method. Three were authored by at least one author of Aigouy et al.^14^ and were set aside. Out of the 10 remaining research articles, 7 also reported using the method. While this is by no means a systematic and generalisable analysis, it is evidence that a biology‐focused article can be cited for its method, and that a method that may initially be designed to answer a specific scientific question can be adapted and reused by others.

## 4 DISCUSSION

Published studies relying on bioimage analysis workflows often fail to provide fully reproducible software pipelines. This concern is supported by broader evidence showing widespread underreporting of imaging methods in biomedical research and persistent challenges in making computational workflows reproducible in the life sciences.^15^, ^16^ This issue has lately been emphasised by the scientific community, which has been actively promoting practices to strengthen reproducibility. Reflecting this collective effort, the NEUBIAS gateway of *F1000Research* ^17^ serves as a hub for knowledge exchange in bioimage analysis. It offers a shared platform for publishing posters, training materials, application‐related and method development articles, perspectives, opinions and reviews on current trends in the field, as well as reports documenting attempts (successful or not) to reproduce bioimage analysis workflows. Within this gateway, code reproducibility is often the main topic being reviewed for both method‐focused articles and reproduction reports. While this is not the standard yet for other journals, it suggests that reviewers of any manuscript based on automated image analysis should be encouraged to assess it through this lens.

Along these lines, other publications highlight that software code must be accompanied by meaningful comments and proper documentation to ensure workflow reproducibility.^3^

Complementing this effort, the publication of checklists for publishing images and image analyses^9^ has also gained considerable attention, as discussed in Section 3.4. Other studies^18^ are beginning to adopt these checklists to enhance the reproducibility of their work, incorporating them as an additional methodological component of their articles.

In view of this literature and following our in‐depth analysis of the archived data, code, and documentation in the original publication by Gehrels et al.,^8^ our assessment is that the software methods and materials provided to allow the reproducibility of the data analysis pipeline associated with the study are, at present, insufficient for independent re‐execution of the bioimage analysis workflow. We do not question the quality of the scientific findings of the original publication; rather, we use the results of our analysis as a clear example of where bioimage analysis workflows can be strengthened to achieve full reproducibility.

Starting with the data, the authors deposited a portion of the dataset in the BioImage Archive (see Section 2.1), which is a valuable effort. However, the available subsets in the archive and the repository are insufficient to execute the workflow, either end‐to‐end or partially, precluding testing of the pipeline stages.

Another positive effort of the original authors is the release of code through a GitHub repository (https://github.com/HiBandan/modelTissueFlow). However, publishing code does not by itself ensure reproducibility. Typically, limitations arise from (i) insufficient documentation and (ii) the absence of an explicit, installable computational environment with pinned dependency versions. Regarding documentation, clear commenting and user‐oriented guidance are crucial for reproducibility.^19^ Code should be understandable to someone outside the original lab. In this case, the repository provides minimal documentation: the code organisation is not clearly described, and the only auxiliary file we found—‘userManual.txt’ (https://github.com/HiBandan/modelTissueFlow/blob/main/examples/userManual.txt)—aims to describe the function of three scripts in approximately five lines of comments. Taken together with the README, this remains quite inadequate, especially if the goal is to support the use of software, which may have required time and resources to be developed, and allowed novel scientific findings. A second issue concerns the software environment specification: while the README explains how to run the code, it does not define versions for dependencies, making installation unreliable. Version conflicts are common and are precisely what containerisation or environment managers (e.g., lockfile‐based tools such as Pixi, https://pixi.sh) are designed to prevent by providing exact, reproducible environments.

Additionally, we note that the GitHub repository has not been updated since the publication of the article. This observation underscores the distinction between releasing code primarily as documentation—which is valuable in its own right^20^—and providing it as a maintained, reusable tool intended to support ongoing community use and development. The responsibility for software maintenance remains a subject of debate, particularly in science where high turnover and a niche market limit long‐term investment. Despite these challenges, version control and quality assurance for open‐source code are firmly established as ‘good scientific practices’.^21^

Another essential aspect for reproducibility concerns the DL component used in one step of the pipeline. Today, several established repositories, such as the BioImage Model Zoo^13^ and Hugging Face,^22^ support the sharing of trained models together with the metadata required for reuse (training details, preprocessing steps, versions). Depositing the trained model in one of these databases would have substantially improved the reproducibility of the methods presented in the analysed publication. The BioImage Model Zoo offers a standardised model format, while Hugging Face is more flexible; depending on technical preferences, either option could have made the AI‐related segment of the workflow readily reusable. Moreover, the availability of public image databases are fundamental for the development of open‐source AI models^23^ and, together with trained DL models, constitute tangible assets that reduce the barriers for the professional development of other researchers, especially early‐career and from countries that lack resources for data acquisition and computational infrastructures.^24^

After a careful review of the original manuscript, supplementary materials, GitHub repository, and BioImage Archive entry, we concluded that achieving a fully reproducible implementation would require input from the authors. We therefore sought their assistance by sending emails to multiple co‐authors and opening an issue in the GitHub repository, but did not receive any response. We also contacted the journal to inquire whether the peer‐review reports could clarify specific points; however, because the reviews are confidential, only the authors could have shared them with us.

We turned to the citing literature to see whether others had mentioned Gehrels et al.’s^8^ bioimage analysis method or used its code. In our experience, it is not uncommon for bioimage analysis workflows to be cited through their associated biology paper,^14^, ^25^ even when they are later published separately.^26^, ^27^ As described in Section 3.5, none of the citing publications explicitly attempted to replicate the analysis or assess the reproducibility of its results. Rather, citations primarily acknowledged the study's contributions or used it as contextual support, without reporting replication efforts or executable confirmations. Although the method may have been designed for a specific research project, bioimage analysis workflows are often complex sequences of steps, modular by nature, that benefit from being both reproducible and reusable. This is true even when they require parameterisation, adaptation, reorganisation, or replacement of some parts to address a different research question. In our opinion, this is the driving force behind initiatives in the bioimage analysis field that publish workflows.^17^, ^28^, ^29^

We do not present the analysed publication as the ‘pathogen’ of poor reproducibility in bioimage analysis, but rather as a potential symptom of broader, systemic factors that can hinder reproducibility. When journals do not incentivise code review and software environment capture, and when authors lack venues or norms for sharing executable workflows, many otherwise solid studies unintentionally become part of the transmission chain. Strengthening the field's ‘immune system’ through publication checklists, model repositories, containerised environments, and methods‐aware peer review, addresses the underlying causes rather than treating a single isolated case.

Meaningful progress will come from a methods‐aware peer review that mandates full data archiving, software environment capture, and AI model deposition. By addressing these underlying causes rather than treating isolated cases, we can ensure that software artefacts are held to the same level of accountability as the biological conclusions they support.

## 5 CONCLUSIONS

In this work, we assessed the reproducibility of the bioimage analysis workflow in a publication by Gehrels et al.^8^ and tried to analyse the availability of documentation, code, and data that are necessary to allow the execution of the data analysis. We focused on the possibility of reproducing the different steps of the workflow, and did not discuss the biological relevance or experimental practices reported by the authors, which have already been approved by expert reviewers in the field.

Following our analysis, we identified key points that made the execution of the workflow difficult for us. These include the lack of clear definitions for annotated regions used to segment or track cell position, the lack of example annotations, the absence of deep learning model weights, and the insufficient amount of deposited data to repeat the analysis.

The dissection of the archived code, and attempts to install and run the software, have highlighted the difficulty of reproducing the analysis of bioimages after only a few years from the publication of major biological studies. In our specific case, the software installation failed across different operating systems, due to the lack of specific versioning for the dependencies, or containerised software environments.

We appreciated that the analysed publication includes a detailed biophysical model and well‐organised code; however, despite the authors' efforts, the currently archived repository does not support end‐to‐end re‐execution of the analysis workflow. This limitation is partly due to the fact that the archived images constitute an example dataset enabling only partial execution of the pipeline.

Screening the citations of the publication under study, we identified how many are referring to the biological findings but none attempted to reproduce the analysis, nor reported using the presented methods or proposed alternatives, at least in published literature. Furthermore, an evaluation of the available code, data, and documentation, using published community checklists, has highlighted consistent reporting gaps, for example, on software versions or how the choice of manual annotations and cell tracking was made.

These gaps in reporting the full methods for bioimage analysis are not a unique trait of the publication under exam, but are rather widely diffused in the bioimaging field.

We hope that the evaluation of this publication against the checklist for publishing new bioimage analysis workflows can convey the important message that minimal fixes, such as the use of pinned environments, the archiving of the full dataset or a portion sufficient to reproduce the bioimage analysis workflow, the release of AI models, and the possibility of running a one‐click demo, can greatly improve the reproducibility of bioimage analysis workflows.

These fixes would benefit both the authors and the scientific community by breaking barriers that currently slow down scientific discovery. Indeed, the confidence that we can have in science is lost when data analysis is difficult to replicate and experiments cannot be reproduced. Improved methods reproducibility and code reusability could also constitute a better base for new projects to build on, and incidentally contribute to diffusing the initial biological findings through citations concerned with the article's methods or code.

With this work, we aim to report a few examples of how a valid scientific work can have an increased impact if the reproducibility of the bioimage analysis workflow is also curated, through well‐documented code and more extensively archived data, thereby empowering other researchers in their scientific endeavour.

## AUTHOR CONTRIBUTIONS

*Conceptualisation*: Mélodie Ambroset, Cesar Augusto Valades Cruz, Rocco D'Antuono, and Caterina Fuster‐Barceló. *Data curation*: Cesar Augusto Valades Cruz and Caterina Fuster‐Barceló. *Citation analysis*: Mélodie Ambroset. *Investigation*: Mélodie Ambroset, Cesar Augusto Valades Cruz, Rocco D'Antuono, and Caterina Fuster‐Barceló. *Project administration*: Caterina Fuster‐Barceló. *Software validation*: Mélodie Ambroset, Cesar Augusto Valades Cruz, Rocco D'Antuono, and Caterina Fuster‐Barceló. *Visualisation*: Mélodie Ambroset, Cesar Augusto Valades Cruz, and Caterina Fuster‐Barceló. *Writing—original draft preparation*: Mélodie Ambroset, Cesar Augusto Valades Cruz, Rocco D'Antuono, and Caterina Fuster‐Barceló. *Writing—review & editing*: Mélodie Ambroset, Cesar Augusto Valades Cruz, Libert Brice Tonfack, Rocco D'Antuono, and Caterina Fuster‐Barceló.

## Supporting information




**Supporting File**: jmi70125‐sup‐0001‐SuppMat.pdf.

## Data Availability

All data and code assessed in this study originate from the original publication. Imaging data are available at the BioImage Archive under accession S‐BIAD602 (https://www.ebi.ac.uk/biostudies/bioimages/studies/S‐BIAD602). The code from Gehrels et al.'s original manuscript is available at https://github.com/HiBandan/modelTissueFlow. The authors of this manuscript did not generate or deposit any new datasets or code.

## Footnotes

1 The publication reports the use of a U‐Net for segmentation without providing specific details regarding its architecture nor parameters. Therefore, the diagram does not represent the exact architecture or training configuration used.
