# Supplementary material for: Assessing the reproducibility of a bioimage analysis workflow characterising tissue flow in Drosophila
Source: J Microsc. 2026 Jun 18;304(2):101–15. doi: 10.1111/jmi.70125 (PMC13686567; doi:10.1111/jmi.70125)
Supplement: Supplementary file 1 [file JMI-304-101-s001.pdf]

Assessing the reproducibility of a bioimage analysis workflow characterising tissue flow in *Drosophila*

Mélodie Ambroset [https://orcid.org/0000-0001-8071-836X][1, 2, 3], Cesar A. Valades Cruz [https://orcid.org/0000-0002-1786-8207][4], Libert Brice Tonfack [https://orcid.org/0000-0002-3478-4187][5], Rocco D'Antuono [https://orcid.org/0000-0003-0180-6500][6, 7, *] and Caterina Fuster-Barceló [https://orcid.org/0000-0002-4784-6957][8, *]

[1] Nantes Université, CHU Nantes, CNRS, INSERM, BioCore, US16, SFR Bonamy, F-44000 Nantes, France
[2] UR BIA, INRAE, Nantes, France
[3] PROBE Research Infrastructure, BIBS Facility, INRAE, Nantes, France
[4] Institute of Hydrobiology, Chinese Academy of Sciences, Wuhan, China
[5] Unit of Plant Physiology and Improvement, Laboratory of Biotechnology and Environment, Department of Plant Biology, Faculty of Science, University of Yaounde 1, Yaounde, Cameroon
[6] Crick Advanced Light Microscopy STP, The Francis Crick Institute, NW1 1AT, London, United Kingdom
[7] Department of Biomedical Engineering, School of Biological Sciences, University of Reading, RG6 6AY, Reading, United Kingdom
[8] BioVisionCenter, Department of Molecular Life Science, Universität Zürich, Zürich, Switzerland

[*] Corresponding authors: rocco.dantuono@crick.ac.uk, caterina.fusterbarcelo@mls.uzh.ch

Supplementary Materials

S.1 Directory tree of the original dataset

```
S-BIAD602
└───DataRepository
    ├───Lightsheet
    │   └───wildtype
    │       └───embryo1_221114_2
    │           └───crop
    │                   221114_GAP43mSc_sqhGFP_raised18_T23_1_5um_2_myo_t10.tif
    │
    └───TwoPhoton
        ├───toll_vl
        │   └───embryo1_210518_2_1
        │       └───MEM
        │               F_000.tif
        │
        └───wildtype
            │   EmbryoInfo210122.rtf
            │
            └───embryo1_210122_2
                │   210122_GAP43mSc_sqhGFP_34_water_2_MEM.tif
                │   210122_GAP43mSc_sqhGFP_34_water_2_MYO.tif
                │
                └───MARKERS
                        F_020.tif
```

Supplementary Figure 1. Directory tree of the S-BIAD602 dataset on BioImage Archive (Gehrels et al. 2022).

S.2 Reproducibility issues on GitHub repository

We tested the installation of the code associated with the analysed manuscript on multiple OS/Python configurations (**Supplementary Table 1**). For each attempt we recorded OS, method, outcome, and errors after some tests. A summary is provided in **Supplementary Table 1**.

**OS Chip**	**OS Version**	**Method**	**Outcome**	**Error headline**
macOS (Apple Silicon)	14.4.1	Git clone	Failed	ImportError: 'gaussian' from scipy.signal
macOS (M4 Max)	15.7.1	pip install modelTissueFlow	Failed	ModuleNotFoundError: No module named 'numpy' (during setup.py egg_info)
macOS (M4 Max)	15.7.1	Git clone	Failed	Undeclared deps cv2, scikit-image; then ImportError: 'gaussian' from scipy.signal
Windows	10.0.19045	pip	Metadata failed	Deprecated “sklearn” meta-package (use scikit-learn)&
Windows	10 Pro	pip	Failed	pip install modelTissueFlow<br>=> Could not import Cython.Distutils. Install `cython` and rerun.<br>pip install cython<br>pip install modelTissueFlow<br>=> The 'sklearn' PyPI package is deprecated, use 'scikit-learn'
Windows	10 Pro	Git clone	Failed	Created env with modified requirements file for scikit-learn, ran analysis.py<br>=> ModuleNotFoundError: No module named 'cv2'<br>pip install opencv-python<br>python analysis.py<br>=> ImportError: cannot import name 'gaussian' from 'scipy.signal'<br>pip install scipy==1.12.0 (downgraded from 1.14.0)<br>python analysis.py<br>=> ModuleNotFoundError: No module named 'shapely'<br>pip install shapely

				python analysis.py<br>=> ModuleNotFoundError: No module named 'openpiv'<br>pip install openpiv<br>=> ImportError: cannot import name 'process' from 'openpiv'

Supplementary Table 1. Summary of the attempts to install the program modelTissueFlow available in the GitHub repository provided by the authors.

As the **Supplementary Table 1** shows, the PyPI path failed during metadata generation because `numpy` is imported at build time but not declared as a build requirement. The source install created import-time failures due to undeclared dependencies `(opencv-python(-headless)`, `scikit-image`) and a SciPy API change (`scipy.signal.gaussian` → `scipy.signal.windows.gaussian`), preventing import in a clean Python 3.10 environment on macOS 15.7.1.

Detailed case study (2025-11-07; macOS 15.7.1, Apple M4 Max)

Environment. Fresh conda env with Python 3.10.

A) **PyPI path**

- Command: `pip install modelTissueFlow`
- Result: Failed (metadata)
- First error: `ModuleNotFoundError: No module named 'numpy'` during python `setup.py egg_info`.
- Interpretation: package imports `numpy` in `setup.py` without declaring it in a modern pyproject.toml build system.

B) **Source path**

1. `git clone …` → `python setup.py install` → Installed (legacy method; deprecation warning).
2. `import modelTissueFlow` → Failed: `ModuleNotFoundError: No module named 'cv2'`.
    a. Fix attempted: `pip install opencv-python-headless`.
3. `import modelTissueFlow` → Failed: `ModuleNotFoundError: No module named 'skimage'`.
    a. Fix attempted: pip install scikit-image.
4. `import modelTissueFlow` → Failed: `ImportError: cannot import name 'gaussian' from scipy.signal`.
    a. Diagnosis: the code uses `from scipy.signal import gaussian`; current SciPy expects `from scipy.signal.windows import gaussian` (or requires pinning SciPy to `<1.11`).

Implication. Even after manual installation of undeclared dependencies, the package remains non-importable without code changes or strict version pinning.

S.3 Citation analysis

Supplementary Table 2. Citation analysis for the original publication (Gehrels et al. 2023)

Ref	Type	Quote	Citation number	Section	Tags
Al-Izzi and Alexander, 2023	Research article	« In many cases these tissues are thin and deformable, and may be modelled as a two-dimensional surface whose active mechanics influences its shape [4–12]. » (Al-Izzi et Alexander, 2023, p. 1)	5	Introduction	General conclusion
Alber et al., 2025	Preprint	« A large body of work has characterized the diverse cellular processes that arise in response to external forces [39], adjacent domains [40, 41], and geometric constraints [42–46], including at the level of individual contributions within a tissue exhibiting both active and passive cellular behaviors [41, 47, 48] » (Alber et al., 2025, p. 2)	46	Introduction	General conclusion
Andrews and Priya, 2025	Review	« In a recent study examining Drosophila gastrulation, the authors used a Fat2 mutant to alter the curvature of the embryo, giving novel insight into how geometry instructs polarized tissue flows (Gehrels et al. 2023). » (Andrews et Priya, 2025, p. 16)	Name	The Issue of Tissue Geometry	General conclusion<br>Theme of the article
Asai et al., 2024	Research article	« Coupling of the large-scale cell flow and body axis morphogenesis has been extensively studied in invertebrate bilaterians, particularly insect models (Keller, 2002; Gehrels et al., 2023; Bertet et al., 2004) » (Asai et al., 2024, p. 2)	Name	Introduction	General conclusion

Bao and Xie, 2023	Research article	« For instance, the curvature of the embryonic tissue can generate differential mechanical tension on cells, which can lead to the spatial arrangement of cells and alterations in gene expression and cell differentiation,15,16 highlighting the importance of geometrical cues in guiding cell differentiation and embryo development. » (Bao et Xie, 2023, p. 34397)	16	Introduction	General conclusion
Biga, Wyatt and Pinheiro, 2023	Meeting Review	« An analysis of tissue flow in the fly embryo by Thomas Lecuit's team uncovered that the geometric coupling between tissue-intrinsic mechanics and a curvature gradient at the posterior pole triggers the onset of polarised flow in this system (Gehrels et al., 2023). » (Biga et al., 2023, p. 3)	Name	The emergence of robust tissue shape	General conclusion
Bouzignac and Suzanne, 2025	Review	« Second, the mismatch between the initial localization of apical Myosin recruitment and the maximum curvature of the embryo contributes to the creation of tissue flow and to the polarization of apical MyoII propagation wave (Fig. 1B) [27 » (Bouzignac et Suzanne, 2025, p. 3)	27	2.1.2. The role of tissue curvature in force propagation	General conclusion
Brauns et al., 2024	Research article	However, the relative contribution of such locally generated forces vs pulling by adjacent tissues, such as the invaginating PMG remain debated. Previous studies addressing this key questions on the tissue level have come to conflicting conclusions (Irvine and Wieschaus, 1994; Streichan et al., 2018; Collinet et al., 2015; Butler et al., 2009; Lye et al., 2015; Farrell et al., 2017; Gehrels et al., 2023).<br><br>« Where the forces driving tissue deformations during morphogenesis	Name	Introduction<br><br>Discussion	General conclusion<br>Theme of the article

		originate is a fundamental question of developmental biology. In the context of Drosophila gastrulation, it has been has been intensively debated whether the germ band elongates due to internally generated stresses (Irvine and Wieschaus, 1994; Bertet et al., 2004; Rauzi et al., 2008; Butler et al., 2009; Gustafson et al., 2022; Streichan et al., 2018) or due to external pulling by the posterior midgut invagination (Collinet et al., 2015; Lye et al., 2015; Bailles et al., 2019; Gehrels et al., 2023). »			
Burda et al., 2023	Review	« In contrast to Drosophila mesoderm invagination, Drosophila endoderm invagination is organized as a propagating spatiotemporal wave of apical constriction that is geometrically coupled with tissue curvature to promote polarized flow.134,135 » (Burda et al., 2023, p. 2859)	135	ILOCAL AND GLOBAL MECHANICAL FEEDBACK REINFORCES PATTERNS OF DIFFERENTIAL GROWTH AND CONTRACTION	General conclusion
Cao et al., 2024	Review	« On the other hand, this tissue-level contractility drives many morphogenesis procedures, such as blastomere compaction,[68] body-axis formation,[69] bending and invaginations,[70] as well as the establishment of segregation or boundaries.[71] » (Cao et al., 2024, p. 3)	71	4. Mechanobiology at the Embryonic Level	General conclusion
Collinet et al., 2024	Research article	« The recruitment of MyoII apically in this highly curved region leads to polarized tissue flow toward the flatter dorsal side of the embryo, where there is a lower cost to bending.44 Thus, both genetic and geometric information underlie the onset of polarized flow.44 » (Collinet et al., 2024, p. 159)<br><br>« Initiation of the wave is determined	44	Introduction Discussion Discussion	General conclusion

		by a genetically controlled invagination in the endoderm primordium35 and by its interplay with egg curvature that gives rise to a polarized flow.44 »<br><br>« The frictional coupling to the vitelline membrane resists the initial anterior flow of the tissue that emerges from tissue curvature44 and thereby contributes to the formation of an invagination by tissue buckling. »			
Cumming and Levayer, 2024	Review	« Similar to local tissue density, curvature can be influenced by multiple factors emerging from local and tissue-wide properties, including fold formation driven by local apical tension increase [86], buckling driven by tissue flow or growth [87,88], folds driven by local modulation of ECM attachment [89,90], differential stiffness of ECM and tissue[91,92], and curvature imposed by the rigid external boundaries of the embryo [93,94], all potentially influencing curvature, extrusion and cell death. » (Cumming et Levayer, 2024, p. 47)	93	1.4. 3D shape and curvature	General conclusion
Dey et al., 2025	Research article	« Recent studies have shown that morphogenetic flow during early development is influenced by the geometry of the boundary.67–69 » (Dey et al., 2025, p. 2297)	67	Discussion	General conclusion
DiFrisco and Priya, 2025	Review	« 1.4 Boundary conditions are physical features or structures that spatially constrain or otherwise limit how a process (such as gene expression or signal diffusion) occurs. For example, curvature, a ubiquitous geometrical property, is known to influence the patterning of mechanical stress, signalling gradients, and to modulate local	72	1. Introduction (Box 1)	Theme of the article

		cellular behaviours [149,155,211,34,72,92]. » (DiFrisco et Priya, 2025, p. 2)			
Erlich and Harmansa, 2025	Review	« Animal cells have the remarkable ability to generate forces in a controlled and active manner. Contractile forces originating from the cell's actomyosin network drive dynamic deformations on the cell and tissue scale [49,90,112] and are well acknowledged in shaping animal tissues (discussed in several excellent reviews [51,62,92]). » (Erlich et Harmansa, 2025, p. 1)	49	Introduction	Theme of the article
Erlich and Zurlo, 2024	Research article	« Note that when we refer to curvature, we do not mean the extrinsic curvature of the actual observed shape (which may guide other morphogenetic processes (Gehrels et al., 2023)), nor do we mean the shape of cells, but rather the intrinsic curvature due to a frustration, or incompatibility, which is present in the material regardless of the deformed body shape. When incompatibility is zero (compatible growth), the body possesses a stress-free configuration as a whole, otherwise multiple (or infinite) cuts are necessary to relax the stresses to zero. » (Erlich et Zurlo, 2024, p. 2)<br><br>« Vertex models have recently received growing attention by the physics community, due to their capability to model phase transitions (jamming/unjamming), where cells can suddenly lose resistance to shear, Farhadifar et al. (2007), Schoetz et al. (2013) and Bi et al. (2015, 2016), but also for being go-to model tools to predict dome formation (Latorre et al., 2018), cell flow in the embryo (Gehrels et al., 2023), intracellular	Name	Introduction	General conclusion

		fluxes in plant tissue (Cheddadi et al., 2019), epithelial folding (Alt et al., 2017). »			
Ghosh et al., 2025	Review	« During embryonic development, similar cell types often assume different geometries [97–99] as tissues undergo dramatic changes in form. » (Ghosh et al., 2025, p. 5)	97	2.2. Force transmission in the nucleus	Theme of the article
Gsell et al., 2025	Researc h article	« Yet, there is growing evidence that tissue mechanics and flows are crucial for many stages of embryonic development17–21. » (Gsell et al., 2025, p. 645)	20	Introduction	General conclusio n
Jie et al., 2025	Preprint	« Given that the tissue geometry has been postulated to pattern 319 mechanical and chemical signals to guide morphogenesis (4, 5, 63-69), we hypothesized that 320 the maximum strain in the outer curvature arises from the anisotropic geometry of myocardial 321 tissue. » (Jie et al., 2025, p. 7)	67	Main	General conclusio n
Ibrahim i and Merkel, 2025	Researc h article	« In many cases, such tissue deformation is at least in part driven by internally generated active stresses [8,10–13], and in recent years, deforming tissues have successfully been described as active materials [8,14–21]. » (Ibrahimi et Merkel, 2025, p. 1)	17	Introduction	General conclusio n
Kingni and Woafo, 2025	Researc h article	« He mentioned that the heredity mechanics is more visible in rubber mechanics, but also in fluid mechanics, in elasticity and in electromagnetism [6–9] » (Kingni et Woafo, 2025, p. 1)	9	Introduction	General conclusio n
Latham et al., 2024	Review	« Finally, polarized tissue flow, such as the anterior movement of the Drosophila endoderm during gastrulation, arises from asymmetrically localized actomyosin activities that produce torque forces	101	1. Cytoskeletal activities	General conclusio n

		along a tissue curvature gradient101. » (Latham et al., 2024, p. 6)			
Manna et al., 2025	Preprint	« In more recent work, researchers have relaxed this condition, allowing frictional or drag forces at the boundary of a tissue to explain tissue morphodynamics.10, 11 » (Manna et al., 2025, p. 2)	11	Introduction	General conclusion
Mishra et al., 2026	Research article	« Recent work using minimal active matter models has revealed that geometry and curvature play a pivotal role in the mechanical response of epithelial sheets1–5. » (Mishra et al., 2026, p. 139)	5	Introduction	General conclusion
Mundhe et al., 2026	Research article	« This induces a polarized flow towards the embryo dorsal-anterior due to the high curvature of the tissue in this region29 (Fig. 1a,b). » (Mundhe et al., 2026, p. 2)	29	Results: GPCR signalling is required in the propagation zone for MyoII activation	General conclusion
Nejad, Yeomans and Thampi, 2025	Preprint	« Therefore there is considerable interest in the biophysics community in applying active matter physics to investigate mechanical and developmental processes in biology [1–5]. Examples include correlated tissue flows in embryogenesis [6, 7], wound healing [8, 9], and cancer development and metastasis [10, 11]. » (Nejad et al., 2025, p. 1)	6	Introduction	Theme of the article
Sabbagh and Harris, 2025	Research article	« Additionally, PMG invagination promotes germband extension by (1) internalizing the posterior columnar epithelium which otherwise blocks germband extension (Sweeton et al., 1991), (2) undergoing columnar-to-wedge cell shape change that aids germband flow around the pole (Gehrels et al., 2023), and (3) generating constriction that pulls germband cells posteriorly (Lye et al., 2015) and promotes germband cell intercalations (Collinet et al., 2015; Yu	Name	Introduction Conclusions	General conclusion

		and Fernandez-Gonzalez, 2016) » (Sabbagh et Harris, 2025, p. 168)<br><br>Although PMG invagination normally pulls the germband (Collinet et al., 2015; Lye et al., 2015) and promotes its flow around the posterior pole (Gehrels et al., 2023), myosin-driven cell intercalation within the germband (Bertet et al., 2004; Blankenship et al., 2006) seems sufficient for germband extension in stepM/Z mutants, and egg shell curvature, atypical PMG invagination, or other dorsal-ventral asymmetries may direct the germband around the pole.			
Serra et al., 2023	Research article	« Theoretical work has also started to elucidate the interplay of signaling, mechanics, and geometrical constraints (31–40) reviewed in (41). » (Serra et al., 2023, p. 2)	39	Introduction	General conclusion
Serrano Nájera et al., 2025	Research article	Morphogenesis requires spatiotemporal coordination of hundreds of thousands of cells to sculpt a developing embryo1,2. Elucidating the principles of morphogenesis, spanning mechanisms from molecular to organismal scales, is a grand challenge for developmental biology and the physics of living systems3–7. Tackling this challenge requires experimental advances and theoretical progress on two fronts: i) developing predictive biophysical models to test hypotheses8–12 and ii) devising mathematical methods to extract key insights from experimental data13–17.	12	Introduction	Theme of the article

Sknepnek et al., 2023	Research article	« This has led to the renewed suggestion that the myosin anisotropy may arise in response to extrinsic forces, such as those generated by the mesoderm invagination of the ventral furrow (Butler et al., 2009), a process that starts somewhat before germband extension and in combination with other extrinsic events such as posterior hindgut invagination, and other geometric constraints could drive germband extension (Collinet et al., 2015; Gehrels et al., 2023). » (Sknepnek et al., 2023, p. 2)	Name	Introduction	General conclusion
Smits et al., 2023	Research article	« Furthermore, it has recently been shown that scb is not important for the initial phases of PMG formation,29 which correlates with when we find the highest degree of asymmetry in wild-type embryos (Figure 4C) » (Smits et al., 2023, p. 3542)	29	Discussion	Result
Talia, 2025	Review	« It has been shown that these models can, in certain circumstances, predict the observed flow patterns (Streichan et al. 2018; Saadaoui et al. 2020), while in other circumstances, flow patterns are also strongly influenced by tissue geometry (Gehrels et al. 2023) and interactions with extraembryonic structures (Münster et al. 2019). » (Talia, 2025, p. 12)	Name	APPLICATIONS OF SIMILAR PRINCIPLES TO OTHER PROBLEMS IN MORPHOGENESIS	General conclusion
Tan et al., 2024	Research article	« Our results are likely to be conserved in rotating spheres derived from other cell types [23,29,30,43], with potential implications for cell migration on complex 3D curved geometries, such as polarized tissue flow in Drosophila melanogaster [44–46]. » (Tan et al., 2024, p. 9)	46	Discussion	Theme of the article

Webb and Sussman, 2025	Research article	« Experimental work has shown that the modulation of both cell shape [26] and motility [27,28] can be mediated by curved surfaces, » (Webb et Sussman, 2025, p. 2)	28	Introduction	General conclusion

Citing references contained in Supplementary Table 2:

Al-Izzi, S.C. and Alexander, G.P. (2023) "Chiral active membranes: Odd mechanics, spontaneous flows, and shape instabilities," Physical Review Research, 5(4), p. 043227. Available at: https://doi.org/10.1103/PhysRevResearch.5.043227.

Alber, D.S. et al. (2025) "A model for boundary-driven tissue morphogenesis." bioRxiv, p. 2025.03.05.641672. Available at: https://doi.org/10.1101/2025.03.05.641672.

Andrews, T.G.R. and Priya, R. (2025) "The Mechanics of Building Functional Organs," Cold Spring Harbor Perspectives in Biology, 17(3), p. a041520. Available at: https://doi.org/10.1101/cshperspect.a041520.

Asai, R. et al. (2024) "Coupling and uncoupling of midline morphogenesis and cell flow in amniote gastrulation," eLife. Edited by R.P. Harvey and S.J. Araújo, 12, p. RP89948. Available at: https://doi.org/10.7554/eLife.89948.

Bao, M. and Xie, J. (2023) "Geometric Confinement-Mediated Mechanical Tension Directs Patterned Differentiation of Mouse ESCs into Organized Germ Layers," ACS Applied Materials & Interfaces, 15(29), pp. 34397–34406. Available at: https://doi.org/10.1021/acsami.3c03798.

Biga, V., Wyatt, T.P.J. and Pinheiro, D. (2023) "Developmental mechanisms understood quantitatively," Development, 150(11), p. dev201727. Available at: https://doi.org/10.1242/dev.201727.

Bouzignac, R. and Suzanne, M. (2025) "Mechanics of force transmission in epithelia: From cell-to-cell propagation to nuclear response," Seminars in Cell & Developmental Biology, 175, p. 103662. Available at: https://doi.org/10.1016/j.semcdb.2025.103662.

Brauns, F. et al. (2024) "The geometric basis of epithelial convergent extension," eLife. Edited by M. Bagnat and F. Campelo, 13, p. RP95521. Available at: https://doi.org/10.7554/eLife.95521.

Burda, I. et al. (2023) “The dynamics and biophysics of shape formation: Common themes in plant and animal morphogenesis,” Developmental Cell, 58(24), pp. 2850–2866. Available at: https://doi.org/10.1016/j.devcel.2023.11.003.

Cao, R. et al. (2024) “A Hierarchical Mechanotransduction System: From Macro to Micro,” Advanced Science, 11(11), p. 2302327. Available at: https://doi.org/10.1002/advs.202302327.

Collinet, C. et al. (2024) “Mechanical regulation of substrate adhesion and de-adhesion drives a cell-contractile wave during Drosophila tissue morphogenesis,” Developmental Cell, 59(1), pp. 156-172.e7. Available at: https://doi.org/10.1016/j.devcel.2023.11.022.

Cumming, T. and Levayer, R. (2024) “Toward a predictive understanding of epithelial cell death,” Seminars in Cell & Developmental Biology, 156, pp. 44–57. Available at: https://doi.org/10.1016/j.semcdb.2023.06.008.

Dey, P. et al. (2025) “Run-and-tumble like motion of a camphor-infused Marangoni swimmer,” Soft Matter, 21(12), pp. 2291–2299. Available at: https://doi.org/10.1039/D4SM01363H.

DiFrisco, J. and Priya, R. (2025) “The interplay of tissue mechanics and gene regulatory networks in the evolution of morphogenesis,” Seminars in Cell & Developmental Biology, 175, p. 103654. Available at: https://doi.org/10.1016/j.semcdb.2025.103654.

Erlich, A. and Harmansa, S. (2025) “How growth-induced stresses guide shape changes during animal morphogenesis: Mechanisms and implications,” Seminars in Cell & Developmental Biology, 175, p. 103661. Available at: https://doi.org/10.1016/j.semcdb.2025.103661.

Erlich, A. and Zurlo, G. (2024) “Incompatibility-driven growth and size control during development,” Journal of the Mechanics and Physics of Solids, 188, p. 105660. Available at: https://doi.org/10.1016/j.jmps.2024.105660.

Ghosh, S. et al. (2025) “Mechanical mechanisms of morphogenesis as potential substrates for evolutionary change,” Seminars in Cell & Developmental Biology, 175, p. 103645. Available at: https://doi.org/10.1016/j.semcdb.2025.103645.

Gsell, S. et al. (2025) “Marangoni-like tissue flows enhance symmetry breaking of embryonic organoids,” Nature Physics, 21(4), pp. 644–653. Available at: https://doi.org/10.1038/s41567-025-02802-2.

Ibrahimi, M. and Merkel, M. (2025) “Stabilization of Active Tissue Deformation by a Dynamic Signaling Gradient,” PRX Life, 3(4), p. 043013. Available at: https://doi.org/10.1103/yq1s-p2zl.

Jie, C.C.J. et al. (2025) “Mechanical fracturing of the extracellular matrix patterns the vertebrate heart.” bioRxiv, p. 2025.03.07.641942. Available at: https://doi.org/10.1101/2025.03.07.641942.

Kingni, S.T. and Woafo, P. (2025) “Effects of Volterra’s formulation of heredity on vibrations in harmonic and Duffing oscillators,” Communications in Nonlinear Science and Numerical Simulation, 146, p. 108799. Available at: https://doi.org/10.1016/j.cnsns.2025.108799.

Latham, Z.D. et al. (2024) “Regulation of epithelial cell jamming transition by cytoskeleton and cell–cell interactions,” Biophysics Reviews, 5(4), p. 041301. Available at: https://doi.org/10.1063/5.0220088.

Manna, R.K. et al. (2025) “Dynamical forces drive cell and organ morphology changes during embryonic development.” bioRxiv, p. 2024.07.13.603371. Available at: https://doi.org/10.1101/2024.07.13.603371.

Mishra, N. et al. (2026) “Geometry-driven asymmetric cell divisions pattern cell cycles and zygotic genome activation in the zebrafish embryo,” Nature Physics, 22(1), pp. 139–150. Available at: https://doi.org/10.1038/s41567-025-03122-1.

Mundhe, G. et al. (2026) “Spatial patterning of contractility by a self-organized mechanogen activity gradient underlies Drosophila gastrulation,” Nature Communications, 17(1), p. 1721. Available at: https://doi.org/10.1038/s41467-026-68418-z.

Nejad, M.R., Yeomans, J.M. and Thampi, S.P. (2025) “Cellular dynamics emerging from turbulent flows steered by active filaments.” arXiv. Available at: https://doi.org/10.48550/arXiv.2501.05971.

Sabbagh, S. and Harris, T.J.C. (2025) “Surrounding tissue morphogenesis with disrupted posterior midgut invagination during Drosophila gastrulation,” Developmental Biology, 517, pp. 168–177. Available at: https://doi.org/10.1016/j.ydbio.2024.10.001.

Serra, M. et al. (2023) “A mechanochemical model recapitulates distinct vertebrate gastrulation modes,” SCIENCE ADVANCES, 9(49). Available at: https://doi.org/10.1126/sciadv.adh8152.

Serrano Nájera, G. et al. (2025) “Control of tissue flows and embryo geometry in avian gastrulation,” Nature Communications, 16(1), p. 5174. Available at: https://doi.org/10.1038/s41467-025-60249-8.

Sknepnek, R. et al. (2023) “Generating active T1 transitions through mechanochemical feedback,” eLife. Edited by K. Kruse and A.M. Walczak, 12, p. e79862. Available at: https://doi.org/10.7554/eLife.79862.

Smits, C.M. et al. (2023) “Maintaining symmetry during body axis elongation,” Current Biology, 33(16), pp. 3536-3543.e6. Available at: https://doi.org/10.1016/j.cub.2023.07.050.

Talia, S.D. (2025) “Developmental Control of Cell Cycle and Signaling,” Cold Spring Harbor Perspectives in Biology, 17(2), p. a041499. Available at: https://doi.org/10.1101/cshperspect.a041499.

Tan, T.H. et al. (2024) “Emergent chirality in active solid rotation of pancreas spheres,” PRX Life, 2(3), p. 033006. Available at: https://doi.org/10.1103/PRXLife.2.033006.

Webb, T.H. and Sussman, D.M. (2025) “curvedSpaceSim: A framework for simulating particles interacting along geodesics,” Computer Physics Communications, 311, p. 109545. Available at: https://doi.org/10.1016/j.cpc.2025.109545.

Supplementary Table 3. Citation analysis of Aigouy et al. 2010 including conference, research and protocol papers published between 2010 and 2013. Papers citing the image analysis method are highlighted in green, or blue for self-citations.

Ref	Type	Quote	Citation number	Cites image analysis method?	Uses software?	Self-citation
Campinho et al., 2013	Research article	« EVL cell division orientation, cell area and orientation of appearing/disappearing junctions were determined using the publicly available Paking Analyser software34 that allows semi-automatic cell segmentation on 2D images (Fig. 1a and Supplementary Video 1). » (Campinho et al., 2013, p. 1415)	34	yes	yes	no

Chacon-Heszele et al., 2012	Research article	« For cellular morphogenesis and long axis quantification, we used Packing Analyzer software (http://idisk-srv1.mpi-cbg.de/~eaton/) (Aigouy et al., 2010) to extract the outline of cells from E14.5 cochlear samples stained with Phalloidin. » (Chacon-Heszele et al., 2012, p. 969)<br>« We extracted cellular outlines (Aigouy et al., 2010) and developed a program to plot the long axis of each cell. » (Chacon-Heszele et al., 2012, p. 972)	Name	yes	yes	no
Giurumescu et al., 2012	Research article	« Despite recent advances, automated segmentation of surface or junction markers remains challenging (Blanchard et al., 2009; Aigouy et al., 2010; Fernandez et al., 2010; Luengo-Oroz et al., 2011); nuclear information can provide a framework for such efforts. » (Giurumescu et al., 2012, p. 4278)	Name	yes	no	no
Hara et al., 2013	Research article	« To quantify the morphology of AM cells, Packing Analyzer V2.0 (Aigouy et al., 2010) was used for the segmentation of cells and measurement of the cell aspect ratio. The segmentation data were imported into ImageJ, and the cell angles were quantified. Rose diagrams were drawn by R software (http://www.r-project.org). » (Hara et al., 2013, p. 484)	Name	yes	yes	no
Ishihara and Sugimura, 2012	Research article	« We approximated an epithelial tissue as a 2-dimensoinal sheet (Aigouy et al., 2011; Bosveld et al., 2012; Farhadifar et al., 2007; Honda, 1983; Rauzi et al., 2008). » (Ishihara et Sugimura, 2012, p. 202)<br>« The accurate detection and tracking of hundreds to thousands of cells in more than 100 sequential images require a very sophisticated algorithm (Aigouy et al., 2011; Bosveld et al., 2012). » (Ishihara et Sugimura, 2012, p. 207)	Name	yes	no	no

Lynch et al., 2013	Research article	« Images were segmented to analyze cell shape using either Packing Analyzer (Aigouy et al., 2010) or SeedWater Segmenter (Mashburn et al., 2012). » (Lynch et al., 2013, p. 207)	Name	yes	yes	no
Mashburn et al., 2012	Research article	« The only currently available tool that is both interactive and capable of segmenting and tracking packed cells in tissue is Packing Analyzer (3); unfortunately, it is still quite slow. » (Mashburn et al., 2012, p. 409)<br>« We also compared the performance of another semiautomatic routine, Packing Analyzer (3), but did not attempt to use its tracking system. » (Mashburn et al., 2012, p. 415)<br>« Before designing our own segmentation tool, we examined several similar tools including ITK-SNAP (12), FIJI's TrakEM2 (9), CellProfiler (4), and Packing Analyzer (3); none of these was able to segment our typical (and thus imperfect) time-lapse image sequences with the speed and accuracy we desired. » (Mashburn et al., 2012, p. 417)<br>« The tool designed to handle image segmentation tasks most similar to ours is Packing Analyzer. It is also a hybrid manual/automatic system (3), but is parameter-based and implements post-process correction and tracking. Packing Analyzer works well with many of our best data sets, but is neither as efficient nor as flexible as needed for our more difficult data sets. » (Mashburn et al., 2012, p. 417)	3	yes	yes	no

Matis, Axelrod and Galic, 2012	Research article	« It is sometimes of interest to monitor cell shape during development (Classen et al., 2005; Aigouy et al., 2010). » (Matis et al., 2012, p. 1307)<br>« A similar method that defines a “magnitude and axis of nematic order for a single cell” and applies it over a field within an image has been described (Aigouy et al., 2010). The output of the method yields data that are similar to a subset of those that our method produces as shown in Figure 4D. One advantage of the Aigouy method is that it maps varying orientation of polarity across an image. However, the Aigouy method was only shown to be suitable for measuring asymmetry of plasma membraneassociated proteins, but not for intracellular proteins, and no statistical analysis of significance is incorporated. » (Matis et al., 2012, p. 1308)	Name	yes	no	no
Warrington, Strutt and Strutt, 2013	Research article	« Cell packing was analysed using Packing Analyzer v2.0 (Aigouy et al., 2010). » (Warrington et al., 2013, p. 1046)<br>« Finally, if planar-polarised distribution of the core proteins is responsible for the asymmetry of E-cad, then we would expect Ecad asymmetry to increase as core protein asymmetry becomes stronger between 20 and 28 hours of pupal life (Aigouy et al., 2010). » (Warrington et al., 2013, p. 1051)<br>« However, during the peak period of junctional rearrangement (from ~18 hours of pupal life) (Classen et al., 2005), the planar-polarised asymmetric distribution of the core proteins is largely lost (Aigouy et al., 2010), and so it might be that during the crucial stage of morphogenesis the core pathway promotes relatively uniform endocytosis of	Name	yes	yes	no

		E-cad. » (Warrington et al., 2013, p. 1053)				
Wu et al., 2013	Research article	« We are grateful to P. Adler, K. Choi, D. Strutt, G. Struhl, J-P. Vincent, S. Cohen, H. Bellen, the Bloomington Drosophila Stock Center and the DSHB for fly strains and reagents, and Benoit Aigouy for providing the 'Packing Analyser V2.0' software. » (Wu et al., 2013, p. 1054)<br>« Polarity as determined from anti-Fmi stained cells was calculated with the software 'packing_analyser_V2' as described in37. The software calculated both angles and strength of polarization (nematic order)37. Nematic order is calculated as the strength (extent) of polarization and angle is calculated as the direction of polarization within a cell. Both measurements are based on the staining intensity around the cell membrane. The detailed method and mathematical formulae used in the calculations are described in ref. 37. » (Wu et al., 2013, p. 1056)<br>« Their expression domain at the wing margin is nearly perpendicular to the initial PCP axes, radial towards the wing margin (Supplementary Fig. S1a; also refs 31,37,38), and also in the eye PCP specification occurs at the stage when R3/R4 are aligned perpendicular to the Wnt-expression domains at the poles of the eye field (Supplementary Fig. S1a', ref. 39), suggesting that they could provide directional cues. » (Wu et al., 2013, p. 1046)<br>« Planar polarization in the wing progressively strengthens during early pupal stages37, suggesting that PCP also requires later input for its strengthening, refinement and maintenance. » (Wu et al., 2013, p. 1046)	37	yes	yes	no

	« Interestingly, cells near the wing margin were oriented parallel to the margin in the double mutant background (for example pink 'nematic order' lines in Fig. 6b"), which is consistent with the proposal that cells in/at the margin respond to cues from the anteroposterior organizer (in third instar stages)31 and are less polarized in wild type at early pupal stages37 (pink 'nematic order' lines in cells in Fig. 6a"c and ref. 37). » (Wu et al., 2013, p. 1050)<br>« The early wing PCP axis (late larval to early pupal stages) correlates well with Wg/dWnt4 margin expression31,37 and, similarly, in the eye polarity is oriented in the dorsoventral axis towards the poles where Wg/Wnt4 are expressed (illustrated in Supplementary Fig. S1a–a'). » (Wu et al., 2013, p. 1051)<br>« polarization strengths (measured by nematic order) in the first few rows of cells near the margin are much weaker than those in cells further away (at 14–17 h APF; ref. 37 » (Wu et al., 2013, p. 1052)<br>« Similarly, PCP orientation in cells near the margin is only very weak early (at 14–16 h APF; ref. 37), » (Wu et al., 2013, p. 1052)<br>« To determine the direct role for Wg/Wnts on Fz–PCP signalling, we examined it at pupal stages, as the patterning role for canonical Wg signalling is much reduced then and PCP still correlates well with Wg/Wnt4 expression (ref. 37 and this study). » (Wu et al., 2013, p. 1052)				
Bai et al., 2013	« "Packing Analyzer"5,6 has been utilized in a number of research investigations that require 2D models of cell boundary networks.7–9 » (Bai et al., 2013, p. 2)	7	yes	yes	yes

Sagner et al., 2012	Research article	« However, we recently identified epithelial morphogenesis as a mechanism that can reorganize global PCP patterns; in the Drosophila pupal wing, oriented cell divisions and rearrangements reorient PCP from a marginoriented pattern to one that points distally [6]. » (Sagner et al., 2012, p. 1296)<br>« We calculated planar cell polarity (PCP) nematics based on Fmi staining [6] and PCP vectors based on the perimeter intensity of EGFP::Pk clones (Figures 1A–1C0; see also Figures S1A and S1B available online). At 72 hr after egg laying (hAEL), the wing pouch has just been specified and is small [7]. » (Sagner et al., 2012, p. 1296)<br>« Nevertheless, most of the PCP defects in ft mutants can be rescued by uniform overexpression of a truncated Ft version that cannot interact with Ds [27, 28], and PCP defects in ds mutants can be rescued by uniform overexpression of Ds [6, 28, 29]. » (Sagner et al., 2012, p. 1300)<br>« Simulating the interplay between PCP and growth in the vertex model showed that oriented cell divisions and cell rearrangements orient PCP either parallel or perpendicular to the axis of tissue elongation, depending on parameters [6]. » (Sagner et al., 2012, p. 1300)<br>« Growth orientation reflects the orientation of both cell divisions and neighbor exchanges, and these can each exert different effects on the axis of PCP [6]. » (Sagner et al., 2012, p. 1300)<br>« We calculated planar cell polarity (PCP) nematics based on Fmi staining [6] and PCP vectors based on the perimeter intensity of EGFP::Pk clones (Figures 1A–1C0; see also Figures S1A and S1B available online). At 72 hr after egg laying (hAEL), the wing pouch has	6	yes	yes	yes

		just been specified and is small [7]. » (Sagner et al., 2012, p. 1296)				
Viktorinová et al., 2011	Research article	« Mathematical models of planar polarization based on complex feedback interaction schemes [7], feedback loops combined with external signalling of stochasticity [8] or mechanical tension [9] have been developed for vector cellular structures like the distally pointing hairs on the Drosophila wing. » (Viktorinová et al., 2011, p. 1059)<br>« The contraction of basal actin filaments could contribute to the elongation of cells that drive the lengthening of the egg chamber along its anteroposterior axis. We therefore used automated image analysis [9] to test for a preferential elongation direction of follicle cells. » (Viktorinová et al., 2011, p. 1059)<br>« Previously, graded molecular cues [18] or directed mechanical stresses [9,19] have been shown to specify global polarity in tissues. » (Viktorinová et al., 2011, p. 1063)	9	yes	yes	yes
Abley et al., 2013	Research article	« At these stages, PCP proteins, such as Vang, already show preferential orientations towards the wing margin (Aigouy et al., 2010). » (Abley et al., 2013, p. 2071)	Name	no		
Bardet et al., 2013	Research article	« Accordingly, quantitative models have analyzed how the adhesion/contractility balance determines junction length, junction tension, and epithelial cell packing (Aigouy et al., 2010; Farhadifar et al., 2007; Graner and Sawada, 1993; Hilgenfeldt et al., 2008; Ka¨fer et al., 2007; Rauzi et al., 2008; Staple et al., 2010). » (Bardet et al., 2013, p. 534)<br>« During Drosophila pupal wing development, cell rearrangements are concomitant to tissue elongation and hexagonal packing formation (Aigouy et al., 2010). The contraction of the proximal wing hinge region triggers wing blade	Name	no		

		elongation and oriented cell rearrangements resulting in a tissue with an elongated shape and regular cell packing at the end of morphogenesis, reminiscent of a honeycomb (Aigouy et al., 2010; Classen et al., 2005). This honeycomb-like pattern formation depends on the activity of the Fat/Dachsous and Frizzled planar cell polarity pathways, both of which regulate cell rearrangements (Aigouy et al., 2010; Bosveld et al., 2012; Classen et al., 2005; Harumoto et al., 2010; Ma et al., 2008; Warrington et al., 2013). » (Bardet et al., 2013, p. 534)<br>« Theoretical models have analyzed how the adhesion/contractility balance determines epithelial cell packing (Aigouy et al., 2010; Farhadifar et al., 2007; Graner and Sawada, 1993; Hilgenfeldt et al., 2008; Ka¨fer et al., 2007; Maıˆtre et al., 2012; Rauzi et al., 2008). » (Bardet et al., 2013, p. 535)<br>« First, we determined that when all sides have the same side parameter value and contractility dominates adhesion (l = lo with 0 < lo < pH, where pH z 3.72 is the normalized perimeter of the regular hexagon), the lattice adopts a honeycomb organization comparable to WT tissue (Aigouy et al., 2010; Farhadifar et al., 2007), with p reaching its minimal value pH (Hales, 2001), homogenous side length l and tension g > 0 (Figures 2A and S2A). » (Bardet et al., 2013, p. 535)<br>and a few others of the same type throughout the article		
Bonnet et al., 2012	Research article	« Several models describe tissues using continuum mechanics [3,6 – 12]. » (Bonnet et al., 2012, p. 1)<br>« The time scales of morphogenesis in this tissue are also of the order of hours [29], as observed in other	12	no

		tissues as well, such as the Drosophila pupal wing [12]. » (Bonnet et al., 2012, p. 6)		
Bosveld et al., 2012	Research article	« The role of the Fat/Ds/Fj pathway in tissue morphogenesis has been studied by using indirect measurements such as the shape of clones and division orientation (19, 20); conversely, measurements of tissue dynamics have so far characterized its role in tissue rotation (21). » (Bosveld et al., 2012, p. 725)	21	no
Brittle, Thomas and Strutt, 2012	Research article	« We also see Dachs asymmetry in 6 hr pupal wings (data not shown) consistent with Ft-Ds-Fj signaling continuing to provide polarizing cues after the third-instar stage [10, 24, 25]. » (Brittle et al., 2012, p. 911)	24	no
Canela-Xandri et al., 2011	Research article	« Related to that, it has been demonstrated in different contexts that the orientation of cell divisions determines the shape of developing tissues and organs [40–45]. » (Canela-Xandri et al., 2011, p. 2)<br>« Nowadays it is widely recognized that in silico experiments are a powerful and effective tool for studying the dynamics of epithelial tissues like the wing imaginal disc [39,45–52]. » (Canela-Xandri et al., 2011, p. 2)<br>« Recent advances include its packing, the AP compartmentalization [39,50], the effects of the mechanical feedback on the tissue topology [52], and the alignment of the planar cell polarity domains with the proximal-distal axis of the wing [45]. » (Canela-Xandri et al., 2011, p. 3)<br>« There are crucial differences in our modeling approach with respect to previous implementations of the vertex model in the context of wing imaginal disc development [39,45,50,52] » (Canela-Xandri et al., 2011, p. 10)	45	no

		« Recent studies suggest that this correlation persists in the wing during all its developmental stages [45,64]. » (Canela-Xandri et al., 2011, p. 11)		
Capilla et al., 2012	Research article	« Some degree of polarization is also evident in prepupal stages (Aigouy et al., 2010; Strutt et al., 2011). » (Capilla et al., 2012, p. 2587)	Name	no
Caruso et al., 2013	Research article	« Here we show that during development, FAT1 controls the shape of subsets of muscles in the facial and scapulohumeral regions, and does so by modulating the polarity of collective myoblast migration, a function in accordance with the emerging link between planar cell polarity and collective directional migration events [29–30,69]. » (Caruso et al., 2013, p. 17)	29	no
Chin & Mlodzik, 2013	Research article	« It has recently been shown that initial polarization of Fz/PCP complexes in Drosophila wings is evident at 5–6 hr APF and even already in late third-instar discs, significantly earlier than the previously assumed 28–30 hr APF stage (Aigouy et al., 2010; Classen et al., 2005; Sagner et al., 2012; Strutt et al., 2011). » (Chin et Mlodzik, 2013, p. 457)<br>« Subsequently, wing cells realign and remodel their PCP along the PD axis, through mechanisms of oriented cell division, cell elongation, cell rotation, and neighbor exchanges (Aigouy et al., 2010). » (Chin et Mlodzik, 2013, p. 457)<br>« Cellular polarization of core PCP factors in developing wings is seen as early as in late third-instar and 5 hr APF stages (Aigouy et al., 2010; Classen et al., 2005; Sagner et al., 2012; Strutt et al., 2011). During early pupal stages, polarization of	Name	no

		core PCP factors is detected in a radial axis (toward wing margin); subsequently, membrane-associated levels of core PCP factors are reduced (during cellular movements caused by the wing hinge contraction, between 18–26 hr APF), and robust core PCP asymmetries "re-emerge" in the proximodistal (P/D) axis at 28 hr APF (Aigouy et al., 2010). To explore the relationship between Fw and Fz, we induced fw LOF clones and analyzed Fz by immunostaining. We detected a reduction of Fz in fw42 mutant clones, most prominently at 22 hr APF (Figures 4A–4B00), the stage when cellular contacts and shapes are rearranged (Aigouy et al., 2010). » (Chin et Mlodzik, 2013, p. 461)		
Daulat et al., 2012	Research article	« Indeed, changes in the localization of PCP proteins that are accompanied by reduced punctum size and the loss of cellular asymmetry (3, 11) occur during junctional remodeling and tissue morphogenesis. » (Daulat et al., 2012, p. 182)	3	no
Devenport et al., 2011	Research article	« However, a recent study of PCP in the dividing Drosophila wing blade also did not report internalization in mitotic cells36. » (Devenport et al., 2011, p. 901)	36	no
Donoughe and DiNardo, 2011	Research article	« Recent work suggests that polarity might be present much earlier during tissue development than had previously been appreciated (Aigouy et al., 2010), but the source of its initial bias remains elusive. » (Donoughe et DiNardo, 2011, p. 2751)	Name	no
Economou et al., 2013	Research article	« Such studies have largely focused on optically accessible non-mammalian species, such as Drosophila (e.g. Butler et al., 2009; Aigouy et al., 2010; Bosveld et al., 2012), zebrafish (e.g. England et al.,	Name	no

		2006; Keller et al., 2008; Blanchard et al., 2009; Olivier et al., 2010), Caenorhabditis elegans (e.g. Bao et al., 2006) and ascidian (e.g. Sherrard et al., 2010). » (Economou et al., 2013, p. 4741)<br>« This has largely been restricted to studies in Drosophila, in which cell shapes are largely based on apical markers rather than full three-dimensional segmentations (Butler et al., 2009; Aigouy et al., 2010; Bosveld et al., 2012). » (Economou et al., 2013, p. 4741)			
Evstifeeva, 2013	Research article	« If a cut is made at the boundary of the reducing distal region of the wing, which prevents the stretching of adjacent regions, the polarity of their cells will not be arranged along the proximodistal axis of the wing and will remain radially oriented as before the reduc tion (Aigouy et al., 2010). » (Evstifeeva, 2013, p. 508)	Name	no	
Fischer et al., 2013	Research article	« Alternatively, work by Aigouy et al. [13] in the Drosophila wing suggests that the global cue is mechanical. They find that, due to the contraction of the hinge, wing cells are subject to anisotropic tension that regulates their alignment. » (Fischer et al., 2013, p. 10)	13	no	
Fürthauer et al., 2012	Research article	« Furthermore, the polarity dynamics of active fluids could be responsible for physiologically relevant structures such as contractile rings in dividing cells [5–7], flows during cellular wound healing [8] or the reorientation of planar cell polarity in tissues [9]. » (Fürthauer et al., 2012, p. 2)	9	no	
Guillot and Lecuit, 2013	Research article	« The loss and gain of adhesive interfaces allows cell intercalation and tissue convergent extension (Bertet et al., 2004; Blankenship et al., 2006) or cell adaptation to tissue stress (by intercalation [Aigouy et al., 2010] and delamination [Eisenhoffer et al., 2012; Marinari et	Name	no	

		al., 2012]). » (Guillot et Lecuit, 2013, p. 227)		
Haigo and Bilder, 2011	Research article	« Engagement of all cells of the tissue in multiple revolutions distinguishes follicle rotation from known phenomena involving partial and local rotation of cell clusters within a tissue (28, 29). » (Haigo et Bilder, 2011, p. 1073)	29	no
Harris et al., 2013	Protocol	« In addition to their roles in resisting mechanical stresses, monolayers are mechanosensitive and can adapt to changes in their mechanical environment through rearrangement of their constituent cells7, extrusion of extranumerary cells8,9 and cell division10. » (Harris et al., 2013, p. 2516)	10	no
Hazelwood and Hancock, 2013	Research article	« This signal or cue that orientates or re-orientates polarity could arise from a morphogen gradient, a gradient of expression or activity of the core PCP proteins [19], or could be due to cellular movement [20], for example. [...] Cellular movement could involve either or both possibilities [20,27]. » (Hazelwood et Hancock, 2013, p. 2)<br>« Alternatively, feedback can also be created by a preferential activation of one intercellular complex over another [20,29,30] and more specifically a preference of Fz-Fmi receptor binding over Vang-Fmi at a particular cell edge [25]. » (Hazelwood et Hancock, 2013, p. 3)<br>« Other proposed mechanisms include hinge contraction and cell flow [20]. » (Hazelwood et Hancock, 2013, p. 12)<br>« An alternative way of describing polarity phenotypes when vector directions are equivalent, for example the equal binding of a protein to each end of a cell, is by using a tensor order parameter as described elsewhere [20]. »	20	no

		(Hazelwood et Hancock, 2013, p. 13)		
Hermle et al., 2013	Research article	« It has also become clear that these PCP domains are already formed in larval stages and are temporarily lost during a phase of junctional remodelling in pupal wing morphogenesis (Aigouy et al, 2010). » (Hermle et al., 2013, p. 245)<br>« PCP domains do exist at this stage, but they are less coherent and point towards the wing margin (Figure 1G and H; Classen et al, 2005; Aigouy et al, 2010). » (Hermle et al., 2013, p. 246)<br>« B) At 18 h APF, VhaPRR localization is more irregular. Junctional staining is still visible, but also intracellular staining, reflecting the dynamic cell rearrangements during this phase (Aigouy et al, 2010). » (Hermle et al., 2013, p. 248)<br>« In immunostainings of pupal wings, we found that VhaPRR co-localized with the PCP domains at all stages of pupal development: in prepupal stages, VhaPRR was polarized towards the wing margin (Figure 2A); during the junctional remodelling phase (Aigouy et al, 2010), the protein partly relocalized to intracellular compartments (Figure 2B);» (Hermle et al., 2013, p. 246)	Name	no
Herszterg et al., 2013	Research article	« In particular, in epithelial tissues, each cell division is found to be associated with the formation of a de novo E-Cad adhesive contact between the daughter cells (BaenaLo´ pez et al., 2005; Gibson et al., 2006; Farhadifar et al., 2007; Aigouy et al., 2010). » (Herszterg et al., 2013, p. 256)<br>« Furthermore, the arrangement of the daughter cells and the geometry of their adhesive contact is important for fundamental aspects of epithelial tissue development,	Name	no

		such as tissue packing and morphogenesis, planar polarization, compactness of somatic clones, and correct signaling between daughter cells (Knox and Brown, 2002; Baena-Lo´ pez et al., 2005; Bo¨ kel et al., 2006; Gibson et al., 2006; Farhadifar et al., 2007; Aigouy et al., 2010). » (Herszterg et al., 2013, p. 256) « The observation of dividing cells in multiple epithelial tissues shows that daughter cells remain in contact upon division (Knox and Brown, 2002; Baena-Lo´ pez et al., 2005; Bo¨kel et al., 2006; Gibson et al., 2006; Aigouy et al., 2010). » (Herszterg et al., 2013, p. 268) « Experimental and theoretical evidence show that this property is essential in numerous contexts, ranging from tissue architecture and planar polarization to cell fate specification (Classen et al., 2005; Gibson et al., 2006; Farhadifar et al., 2007; Aigouy et al., 2010) » (Herszterg et al., 2013, p. 268)		
Hogan et al., 2011	Research article	« In addition, recently published work from the Eaton lab has also identified the L3 vein as the boundary between oppositely polarized cells in the anterior and posterior of early pupal wings [40]. » (Hogan et al., 2011, p. 10) « Significantly, the Eaton lab has recently shown that the subcellular localization of Vang/Stbm protein in the early (15 hours a.p.f.) pupal wing of a ds mutant is more distal than wild-type in both the anterior and distal posterior wing (see Figure 7C in [40]). » (Hogan et al., 2011, p. 10)	40	no
Jieling Zhao et al., 2013	Conference article	« Vertex models are another group of widely used models [10]–[12], [16] » (Jieling Zhao et al., 2013, p. 4517)	12	no

Kennaway et al., 2011	Research article	« At the tissue scale it may therefore be reasonable to model many animal structures with the framework described here, particularly as orientated cell behaviours are thought to play a critical role [39,40]. » (Kennaway et al., 2011, p. 11)	40	no
Lawlor, Ly and DiNardo, 2013	Research article	« Furthermore, new analysis suggests that the initial polarization events need to occur only over a small region comprising few cell widths. Once set, computer modeling combined with experimental evidence suggests that this incipient polarization can be grown through morphogenesis, rather than continually developed and re-inforced by long range gradients (Matakatsu and Blair, 2004; Ma et al., 2008; Aiguoy et al., 2010; Sagner et al., 2012). » (Lawlor et al., 2013, p. 286)<br>« Recent work in discs suggests that polarization needs to occur only over a few cell widths, and that, once established, this incipient polarization can be grown through morphogenesis, rather than continually developed by long range gradients (Matakatsu and Blair, 2004; Ma et al., 2008; Aiguoy et al., 2010; Sagner et al., 2012). » (Lawlor et al., 2013, p. 293)	Name	no
LeGoff, Rouault and Lecuit, 2013	Research article	« Although mechanical stress has been shown to play a role in a range of morphogenetic processes such as gastrulation (Butler et al., 2009; Rauzi et al., 2008) or planar cell polarity (PCP) (Aigouy et al., 2010; Olguín et al., 2011), the context of a growing wing disc raises a new question: how do the constant cell rearrangements provided by cell divisions affect the distribution of stress in the tissue? » (LeGoff et al., 2013, p. 4056)	Name	no

Lewellyn, Cetera and Horne-Badovinac, 2013	Research article	« Recent studies have shown that forces exerted by epithelial morphogenesis or by tension from neighboring tissues can align the Frizzled/Stabismus planar cell polarity system (Aigouy et al., 2010; Olguín et al., 2011; Lee et al., 2012). » (Lewellyn et al., 2013, p. 727)	Name	no
Li et al., 2012	Research article	« The oriented tension is also found during tissue elongation in Drosophila [40]. » (Li et al., 2012, p. 7)	40	no
Mao et al., 2013	Research article	« Morphogenesis then further sculpts this post-growth tissue mass through cell-shape changes and cell rearrangements (Lecuit and Le Goff, 2007; Keller et al, 2008; Aigouy et al, 2010; Bosveld et al, 2012) to produce a tissue of the correct final shape. » (Mao et al., 2013, p. 2790)	Name	no
Marcinkevicius and Zallen, 2013	Research article	« Fat and Dachsous regulate cell shape, rearrangement and oriented divisions during tissue elongation in the Drosophila wing (Baena-López et al., 2005; Aigouy et al., 2010), and mammalian Fat4 and Dachsous 1 are required for elongation of the neural tube, cochlea, kidney and intestines (Saburi et al., 2008; Saburi et al., 2012; Mao et al., 2011a) » (Marcinkevicius et Zallen, 2013, p. 433)<br>« Fat/Dachsous signaling is required for polarized cell division and cell rearrangements in Drosophila (Baena-López et al., 2005; Aigouy et al., 2010; Bosveld et al., 2012), and for tissue elongation in the mouse neural tube, cochlea, kidney and intestines (Saburi et al., 2008; Saburi et al., 2012; Mao et al., 2011a). » (Marcinkevicius et Zallen, 2013, p. 441)	Name	no

Márquez et al., 2012	Research article	« Ultimately by tailoring our interpolation models to experimentally observed developmental changes in shape, for example through spatial heterogeneity of growth rates under mechanical stress (Rauzi et al. 2008; Aigouy et al. 2010), we can hope to infer something about the processes that give rise to a more general set of changes in form. » (Márquez et al., 2012, p. 420)<br>« The fact that many of the experimentally-based attempts to link development and form mathematically are based on biomechanical and mechanochemical properties of viscoelastic fields undergoing stress (reviewed by Humphrey 2003; Davidson et al. 2009; Roeder et al. 2011), suggests ample common ground to pursue unitary models to explain both patterns and mechanisms underlying shape variation. Some examples include the organizing role of anisotropic cortical strain distributions on planar cell polarity (e.g., Aigouy et al. 2010), cell and tissue deformation (e.g., Rauzi et al. 2008; Blanchard et al. 2009; Rauzi and Lenne 2011), growth (e.g., Nelson et al. 2005; Shraiman 2005), and epithelial packing (Farhadifar et al. 2007; Aegerter-Wilmsen et al. 2010). » (Márquez et al., 2012, p. 433)	Name	no
Matakatsu and Blair, 2012	Research article	« This is also in agreement with the substantial improvement of ds and ds fj mutant PCP defects by uniformly expressed ds and fj (Matakatsu and Blair, 2004; Simon, 2004; Aigouy et al., 2010). One hypothesis suggests that the adhesion mediated by uniform Ds provides tension during elongation of the pupal wing blade, aiding in the reorientation of cell polarity along the proximo-distal axis of the wing	Name	no

		(Aigouy et al., 2010).» (Matakatsu et Blair, 2012, p. 1505)			
Mirkovic, Pylawka and Hudspeth, 2012	Research article	« Although the asymmetric subcellular localization of PCP components ensures the directional propagation of polarity signals, it remains unclear how asymmetry is established initially and how cells interpret polarity information (Wu and Mlodzik, 2009; Aigouy et al., 2010). » (Mirkovic et al., 2012, p. 498)	Name	no	
Morishita and Hironaka, 2013	Research article	« This force induces global and dynamic cell rearrangement through cell elongation, cell division and cell migration, resulting in global orientation of PCP [59]. » (Morishita et Hironaka, 2013, p. 48)	59	no	
Mouri, Horiuchi and Uemura, 2012	Research article	« The increase in the Fmi level became stronger and more pronounced between 20 and 32 h APF, and this time period includes a number of cellular dynamic changes: around 24 h, cell proliferation ceases and cells elongate most extremely along the P-D axis, which is concomitant with P-D alignment of microtubules; also in this 20–32 h APF time window, cells continuously rearrange, leading to hexagonal packing (Aigouy et al. 2010; Harumoto et al. 2010). » (Mouri et al., 2012, p. 520)	Name	no	
Noël et al., 2013	Research article	« The mechanism by which such cells acquire polarized actomyosin activity remains unclear, although it is also possible that tissue tension within the heart, driven by addition of cells from the second heart field to the growing heart tube, orients actin localization or myosin recruitment similar to the orientation of tissue polarity by cellular flow in Drosophila axis and wing elongation50,51. » (Noël et al., 2013, p. 7)	51	no	

Olguín, Glavic and Mlodzik, 2011	Research article	« The fat (ft)/dachsous (ds) system controls PCP establishment in parallel to Fz/PCP signaling [14], so we tested whether chas works through this system. The nota of strong ds combinations (ds38k/dsUA071) or pnrG4-driven ftiR, dsiR, display bristles that are slightly oriented laterally (Figure 2D; Figure S2), likely related to their mild thorax cleft and shape and size defects (consistent with one or more roles in tissue growth and cell behavior [15]). » (Olguín et al., 2011, p. 237)	15	no
Pan et al., 2013	Research article	« Wing elongation is influenced by Fat-PCP signaling both during disc growth, when it polarizes cell divisions along the proximal-distal axis, and during pupal development, when it influences local cell rearrangements (Baena-López et al., 2005; Aigouy et al., 2010; Mao et al., 2011a). » (Pan et al., 2013, p. 833)	Name	no
Panousopoulou et al., 2013	Research article	« A similar role for physical force has been reported for the fly wing blade hinge contraction that orients cell polarity over long distances (Aigouy et al., 2010). » (Panousopoulou et al., 2013, p. 501)	Name	no
Petrov and Aegerter, 2011	Research article	« The experimentally observed types of spatial patterns of Drosophila wing structures present regular lattices (for example — hexagonal distributions) of cells31,32 or irregular ones. » (Petrov et Aegerter, 2011, p. 730)	31	no
Rolfe, Roddy and Murphy, 2013	Research article	« Several recent studies on Drosophila development have shown the importance of mechanical signals in cell polarization, in particular impacting the planar cell polarity (PCP) pathway, which shares components of the Wnt signaling pathway [7, 8]. » (Rolfe et al., 2013, p. 108)<br>« Although much remains to be elucidated, it is clear that	7	no

		mechanical signals impact classic developmental signaling pathways including Wnt [7, 8, 52], can be sensed through cytoskeletal tension, possibly through integrins and deformation of other ECM components [53–56], and is often mediated by the Rho-Rock/ MyoII pathway [5]. » (Rolfe et al., 2013, p. 112)		
Salbreux et al., 2012	Research article	« Our model assumes that the edges are circular arcs, as they must be in mechanical equilibrium if the cells possess an isotropic pressure conjugate to their (two-dimensional) area and the cell-cell interfaces do not resist bending [66]; more generally, this assumption represents the simplest possible extension of the widely-used vertex models, which enforce straight edges [37,60,62,67], to allow for the significant interfacial curvature seen in some of our images of fish retina. » (Salbreux et al., 2012, p. 7)<br>« The model also includes a schematic description of PCP loosely based on what is known about Drosophila wing imaginal discs, but flexible enough to allow for alternative molecular mechanisms that also lead to polarization [37,41] » (Salbreux et al., 2012, p. 7)<br>« Such effective energy models have been quite successful in describing cell shape in other epithelia [37,6061,63,72–73]. » (Salbreux et al., 2012, p. 8)<br>« As just described, our model is based on two effective energies: a mechanical energy Fm and an energy Fp governing the dynamics of polarity proteins (compare [37]). » (Salbreux et al., 2012, p. 8)"	37	no
Sharma and McNeill, 2013	Research	« The relationship between The Ft-Ds and Fz/PCP pathways is complex and context dependent	Name	no

	article	(Adler et al., 1998; Aigouy et al., 2010; Casal et al., 2006; Hogan et al., 2011; Ma et al., 2003; Matakatsu and Blair, 2004; Sagner et al., 2012; Strutt and Strutt, 2002; Yang et al., 2002). » (Sharma et McNeill, 2013, p. 3877)		
Shwartz et al., 2012	Research article	« A logical extension of this notion is the expected crossspecies conservation of this mechanism. Interestingly, recent studies in drosophila link mechanical signals to cell polarization, which is a prerequisite for proper intercalation (Aigouy et al., 2010; Olguin et al., 2011). » (Shwartz et al., 2012, p. 161)	Name	no
Sienknecht et al., 2011	Research article	« An analogous reorientation process occurs in epithelium of the fly wing. There, reorientation was recently shown to be associated with polarized axes of cell division, orientated cellular flow leading to elongation and narrowing of the wing primordium, and rearrangements of neighboring cells that is accompanied by a biased orientation of new vs. stable cell boundaries marked by Vangl2 expression (Aigouy et al., 2010). » (Sienknecht et al., 2011, p. 37)<br>« Interestingly, however, recent analyses of Drosophila wing development suggest that no long-range signaling is necessary to establish and maintain global polarity and that a coordinating role of the Ft/Ds pathway in the fly is consistent even when the polarity vectors change during development as reorientation takes place (Aigouy et al., 2010). » (Sienknecht et al., 2011, p. 38)	Name	no

Staple et al., 2010	Research article	« A physical description of tissue organization and mechanics can thus be based on the mechanics of the junctional network [3]. » (Staple et al., 2010, p. 117)<br>« We have recently presented a vertex model that can account for the geometry of the junctional network in the wing disc (epithelium that forms the developing wing) of the fruit fly Drosophila [3]. » (Staple et al., 2010, p. 118)<br>« Our results extend and refine those presented in [3]. » (Staple et al., 2010, p. 118)<br>« However, these configurations are force-balanced, and thus local minima of the potential F [3]. » (Staple et al., 2010, p. 121)<br>« In the vertex model, cell division can be introduced by the following steps [3] » (Staple et al., 2010, p. 122)<br>« This data is compared to experimental estimates of neighbor number distributions in the developing wing of fruit fly Drosophila (data from ref. [3]). » (Staple et al., 2010, p. 123)<br>« In ref. [3], a vertex model was shown to reproduce morphology and topology of epithelial network configurations in the developing wing of the fruit fly. » (Staple et al., 2010, p. 123)<br>« Method B is the algorithm used in ref. [3]. » (Staple et al., 2010, p. 126)<br>« Algorithm II for quasistatic cell division introduced in this paper is a simplified version of Algorithm I, which was used in ref. [3], see sect. 6.1.» (Staple et al., 2010, p. 126)<br>« Note that the polygon distributions shown in ref. [3] differ slightly from the dashed line in fig. 8, which is obtained using the same algorithm. Reasons for this are: i) A length cutoff was used when defining neighbour numbers in ref. [3], which is necessary when comparing with	3	no	no

		experiment. No such cutoff is used here. ii) In ref. [3] a single network containing 104 cells was used, whereas here we use 103 networks (dashed line in fig. 8) and 104 networks (solid line) containing ~ 110 cells each. This implies a larger statistical error in ref. [3] compared to fig. 8. iii) There are additional numerical inaccuracies in ref. [3]. » (Staple et al., 2010, p. 126)		
Strutt, Warrington and Strutt, 2011	Research article	« In the Drosophila early pupal wing, asymmetric localization is seen within the adherens junction region, oriented toward the wing margin (Classen et al., 2005; Aigouy et al., 2010). Cell rearrangements then lead to a remodeling of asymmetry, such that it aligns on the proximodistal (PD) axis (Aigouy et al., 2010). » (Strutt et al., 2011, p. 511)<br>« The core proteins are most strongly asymmetrically localized just before trichome initiation at around 28 hr after prepupa formation (APF) (Strutt and Strutt, 2009; Aigouy et al., 2010) » (Strutt et al., 2011, p. 513)<br>« Asymmetric localization of the core proteins is seen at this stage (Figures 1C, 1E, and 1F; see also Classen et al., 2005; Aigouy et al., 2010), although it is less coherent than at 28 hr and oriented toward the wing margin. » (Strutt et al., 2011, p. 513)<br>« High-resolution images of 28-hr pupal wings showed that at this stage, proximally and distally localizing polarity proteins were also concentrated in puncta in PD junctions (Figures 3D3F; see also Aigouy et al., 2010). » (Strutt et al., 2011, p. 515)<br>« However, at 20 hr APF, when cells are undergoing junctional remodeling, asymmetric localization is poor (Classen et al., 2005; Aigouy	Name	no

		et al., 2010), and puncta are smaller than at 28 hr (Figure 3Q). » (Strutt et al., 2011, p. 515)<br>« Our time-lapse experiments indicate that individual puncta are stable for several hours (see also Aigouy et al., 2010). Nevertheless, in the Drosophila wing, morphogenetic changes such as wing eversion, hinge contraction, and junctional remodeling (Classen et al., 2005; Aigouy et al., 2010) necessitate some rearrangement of junctions, and this appears to be accompanied by reduced puncta size and loss of cellular asymmetry. » (Strutt et al., 2011, p. 523)		
Sugimura and Ishihara, 2013	Research article	« Cell geometry (e.g. the eventual fraction of hexagonal cells) is highly reproducible in each epithelial tissue and is regulated by forces acting along the plane of the adherens junction, such as tension, which shortens a cell contact surface, and pressure, which counteracts tension to maintain the size of a cell (Fig. 1A) (Graner and Sawada, 1993; Mofrad and Kamm, 2006; Farhadifar et al., 2007; Käfer et al., 2007; Lecuit and Lenne, 2007; Honda et al., 2008; Rauzi et al., 2008; Aigouy et al., 2010; Dahmann et al., 2011; Eaton and Jülicher, 2011; Lecuit et al., 2011; Kasza and Zallen, 2011; Bosveld et al., 2012; Sano et al., 2012). » (Sugimura et Ishihara, 2013, p. 4091)<br>« In phase I [from ~15-24 hours after puparium formation (APF)] of wing development, cells undergo one or two rounds of cell division, and the initial, nearly isotropic morphology of the cells becomes elongated along the proximaldistal (PD) axis of the wing (Fig. 1C) (Fristrom and Fristrom, 1993; Classen et al., 2005; Aigouy et al., 2010). I » (Sugimura et Ishihara, 2013, p. 4091)	Name	no

		« Studies based on sophisticated image analyses have clarified the regulation of cell rearrangement by planar cell polarity (PCP) proteins (Strutt and Strutt, 2009), Rap1 GTPase (Knox and Brown, 2002) and tissue stretching along the PD axis by hinge constriction (Classen et al., 2005; Aigouy et al., 2010; O'Keefe et al., 2012). » (Sugimura et Ishihara, 2013, p. 4091)<br>« C) Cell-level dynamics in the Drosophila pupal wing (Aigouy et al., 2010). Wing cells at each stage are colored according to their number of edges (red, square; green, pentagon; gray, hexagon; blue, heptagon; magenta, octagon). » (Sugimura et Ishihara, 2013, p. 4092)<br>« To test the possibility that anisotropic tissue stress plays an important role in hexagonal pattern formation, the wing was detached with the hinge by forceps at 23.5-24 hours APF, as previously described (Aigouy et al., 2010). » (Sugimura et Ishihara, 2013, p. 4093)<br>« Consistent with this hypothesis, a decrease in the number of hexagonal cells was observed by severing the wing from the hinge at an earlier developmental stage (Aigouy et al., 2010); however, further studies are necessary because pleiotropic effects, such as alternation of cell division patterns, are also induced by this technique (Aigouy et al., 2010; data not shown).» (Sugimura et Ishihara, 2013, p. 4095-4096)		
Sugimura et al., 2013	Conference article	« Quantitative data of cell-level dynamics in the wing suggests that forces change significantly over hours [6]. » (Sugimura et al., 2013, p. 2713)<br>« STP and ST agree in the determination of stress in Fig. 4, STP being more robust. Then SP may underestimate it. This is	6	no

		because stronger tensions on the proximal-distal edges of the wing [6], [15] were not incorporated in SP. » (Sugimura et al., 2013, p. 2715)		
Vasilica Stirbat et al., 2013	Research article	« Morphogenetic processes involve cell flows, and developing tissues have been shown to display a fluid-like behavior at large timescales [1,2]. » (Vasilica Stirbat et al., 2013, p. 1)	1	no
Wibowo et al., 2011	Research article	« It may reveal a general process that links epithelial polarity to external mechanical cues (Aigouy et al., 2010). » (Wibowo et al., 2011, p. 1152)	Name	no
Zhu and Owen, 2013	Research article	« The concise model thus contains four key components - Fz, Dsh, Stbm, and Pk as representatives of distal transmembrane and cytoplasmic components and proximal transmembrane and cytoplasmic components. Similar to previous models28,29, an epithelial cell was divided into six compartments, the amount of each molecule was conserved in each cell during the simulated developmental period, and the model simulated cell polarization under cues during a limited time period without considering PCP's global reorientation38. » (Zhu et Owen, 2013, p. 2)<br>« The global PCP pattern in the wing is gradually reoriented toward the distal side38. » (Zhu et Owen, 2013, p. 8)	38	no
Žigman et al., 2011	Research article	« The only similar feedback loop to our knowledge between cell shape and cytoskeletal organization for mitosis in vivo has been proposed for fission yeast [39], and very recently for cell orientation by anisotropic tension in the Drosophila pupal wing epithelium [40]. » (Žigman et al., 2011, p. 85)	40	no

Citing references contained in Supplementary Table 3:

Abley, K. *et al.* (2013) “An intracellular partitioning-based framework for tissue cell polarity in plants and animals,” *Development*, 140(10), pp. 2061–2074. Available at: https://doi.org/10.1242/dev.062984.

Bai, L. *et al.* (2013) “3D surface reconstruction and visualization of the *Drosophila* wing imaginal disc at cellular resolution,” in P.C. Wong et al. (eds.). *IS&T/SPIE Electronic Imaging*, Burlingame, California, USA, p. 86540D. Available at: https://doi.org/10.1117/12.2008497.

Bardet, P.-L. *et al.* (2013) “PTEN Controls Junction Lengthening and Stability during Cell Rearrangement in Epithelial Tissue,” *Developmental Cell*, 25(5), pp. 534–546. Available at: https://doi.org/10.1016/j.devcel.2013.04.020.

Bonnet, I. *et al.* (2012) “Mechanical state, material properties and continuous description of an epithelial tissue,” *Journal of The Royal Society Interface*, 9(75), pp. 2614–2623. Available at: https://doi.org/10.1098/rsif.2012.0263.

Bosveld, F. *et al.* (2012) “Mechanical Control of Morphogenesis by Fat/Dachsous/Four-Jointed Planar Cell Polarity Pathway,” *Science*, 336(6082), pp. 724–727. Available at: https://doi.org/10.1126/science.1221071.

Brittle, A., Thomas, C. and Strutt, D. (2012) “Planar Polarity Specification through Asymmetric Subcellular Localization of Fat and Dachsous,” *Current Biology*, 22(10), pp. 907–914. Available at: https://doi.org/10.1016/j.cub.2012.03.053.

Campinho, P. *et al.* (2013) “Tension-oriented cell divisions limit anisotropic tissue tension in epithelial spreading during zebrafish epiboly,” *Nature Cell Biology*, 15(12), pp. 1405–1414. Available at: https://doi.org/10.1038/ncb2869.

Canela-Xandri, O. *et al.* (2011) “Dynamics and Mechanical Stability of the Developing Dorsoventral Organizer of the Wing Imaginal Disc,” *PLOS Computational Biology*, 7(9), p. e1002153. Available at: https://doi.org/10.1371/journal.pcbi.1002153.

Capilla, A. *et al.* (2012) “Planar cell polarity controls directional Notch signaling in the Drosophila leg,” *Development*, 139(14), pp. 2584–2593. Available at: https://doi.org/10.1242/dev.077446.

Caruso, N. *et al.* (2013) “Deregulation of the Protocadherin Gene FAT1 Alters Muscle Shapes: Implications for the Pathogenesis of Facioscapulohumeral Dystrophy,”

PLOS Genetics, 9(6), p. e1003550. Available at: https://doi.org/10.1371/journal.pgen.1003550.

Chacon-Heszele, M.F. *et al.* (2012) "Regulation of cochlear convergent extension by the vertebrate planar cell polarity pathway is dependent on p120-catenin," *Development*, 139(5), pp. 968–978. Available at: https://doi.org/10.1242/dev.065326.

Chin, M.-L. and Mlodzik, M. (2013) "The Drosophila Selectin Furrowed Mediates Intercellular Planar Cell Polarity Interactions via Frizzled Stabilization," *Developmental Cell*, 26(5), pp. 455–468. Available at: https://doi.org/10.1016/j.devcel.2013.07.006.

Daulat, A.M. *et al.* (2012) "Mink1 Regulates β-Catenin-Independent Wnt Signaling via Prickle Phosphorylation," *Molecular and Cellular Biology*, 32(1), pp. 173–185. Available at: https://doi.org/10.1128/MCB.06320-11.

Devenport, D. *et al.* (2011) "Mitotic internalization of planar cell polarity proteins preserves tissue polarity," *Nature Cell Biology*, 13(8), pp. 893–902. Available at: https://doi.org/10.1038/ncb2284.

Donoughe, S. and DiNardo, S. (2011) "dachsous and frizzled contribute separately to planar polarity in the Drosophila ventral epidermis," *Development*, 138(13), pp. 2751–2759. Available at: https://doi.org/10.1242/dev.063024.

Economou, A.D. *et al.* (2013) "Whole population cell analysis of a landmark-rich mammalian epithelium reveals multiple elongation mechanisms," *Development*, 140(23), pp. 4740–4750. Available at: https://doi.org/10.1242/dev.096545.

Evstifeeva, A.Yu. (2013) "Topology and planar polarity of the Xenopus embryonic ciliated epithelium," *Biology Bulletin*, 40(6), pp. 508–514. Available at: https://doi.org/10.1134/S1062359013060046.

Fischer, S. *et al.* (2013) "Is a Persistent Global Bias Necessary for the Establishment of Planar Cell Polarity?," *PLOS ONE*, 8(4), p. e60064. Available at: https://doi.org/10.1371/journal.pone.0060064.

Fürthauer, S. *et al.* (2012) "The Taylor–Couette motor: spontaneous flows of active polar fluids between two coaxial cylinders," *New Journal of Physics*, 14(2), p. 023001. Available at: https://doi.org/10.1088/1367-2630/14/2/023001.

Giurumescu, C.A. *et al.* (2012) “Quantitative semi-automated analysis of morphogenesis with single-cell resolution in complex embryos,” *Development*, 139(22), pp. 4271–4279. Available at: https://doi.org/10.1242/dev.086256.

Guillot, C. and Lecuit, T. (2013) “Adhesion Disengagement Uncouples Intrinsic and Extrinsic Forces to Drive Cytokinesis in Epithelial Tissues,” *Developmental Cell*, 24(3), pp. 227–241. Available at: https://doi.org/10.1016/j.devcel.2013.01.010.

Haigo, S.L. and Bilder, D. (2011) “Global Tissue Revolutions in a Morphogenetic Movement Controlling Elongation,” *Science*, 331(6020), pp. 1071–1074. Available at: https://doi.org/10.1126/science.1199424.

Hara, Y. *et al.* (2013) “Directional migration of leading-edge mesoderm generates physical forces: Implication in *Xenopus* notochord formation during gastrulation,” *Developmental Biology*, 382(2), pp. 482–495. Available at: https://doi.org/10.1016/j.ydbio.2013.07.023.

Harris, A.R. *et al.* (2013) “Generating suspended cell monolayers for mechanobiological studies,” *Nature Protocols*, 8(12), pp. 2516–2530. Available at: https://doi.org/10.1038/nprot.2013.151.

Hazelwood, L.D. and Hancock, J.M. (2013) “Functional modelling of planar cell polarity: an approach for identifying molecular function,” *BMC Developmental Biology*, 13(1), p. 20. Available at: https://doi.org/10.1186/1471-213X-13-20.

Hermle, T. *et al.* (2013) “Drosophila ATP6AP2/VhaPRR functions both as a novel planar cell polarity core protein and a regulator of endosomal trafficking,” *The EMBO Journal*, 32(2), pp. 245–259. Available at: https://doi.org/10.1038/emboj.2012.323.

Herszterg, S. *et al.* (2013) “Interplay between the Dividing Cell and Its Neighbors Regulates Adherens Junction Formation during Cytokinesis in Epithelial Tissue,” *Developmental Cell*, 24(3), pp. 256–270. Available at: https://doi.org/10.1016/j.devcel.2012.11.019.

Hogan, J. *et al.* (2011) “Two Frizzled Planar Cell Polarity Signals in the Drosophila Wing Are Differentially Organized by the Fat/Dachsous Pathway,” *PLOS Genetics*, 7(2), p. e1001305. Available at: https://doi.org/10.1371/journal.pgen.1001305.

Ishihara, S. and Sugimura, K. (2012) “Bayesian inference of force dynamics during morphogenesis,” *Journal of Theoretical Biology*, 313, pp. 201–211. Available at: https://doi.org/10.1016/j.jtbi.2012.08.017.

Jieling Zhao *et al.* (2013) “Dynamic mechanical finite element model of biological cells for studying cellular pattern formation,” *2013 35th Annual International Conference of the IEEE Engineering in Medicine and Biology Society (EMBC)*. *2013 35th Annual International Conference of the IEEE Engineering in Medicine and Biology Society (EMBC)*, Osaka: IEEE, pp. 4517–4520. Available at: https://doi.org/10.1109/EMBC.2013.6610551.

Kennaway, R. *et al.* (2011) “Generation of Diverse Biological Forms through Combinatorial Interactions between Tissue Polarity and Growth,” *PLOS Computational Biology*, 7(6), p. e1002071. Available at: https://doi.org/10.1371/journal.pcbi.1002071.

Lawlor, K.T., Ly, D.C. and DiNardo, S. (2013) “*Drosophila* Dachsous and Fat polarize actin-based protrusions over a restricted domain of the embryonic denticle field,” *Developmental Biology*, 383(2), pp. 285–294. Available at: https://doi.org/10.1016/j.ydbio.2013.09.007.

LeGoff, L., Rouault, H. and Lecuit, T. (2013) “A global pattern of mechanical stress polarizes cell divisions and cell shape in the growing Drosophila wing disc,” *Development*, 140(19), pp. 4051–4059. Available at: https://doi.org/10.1242/dev.090878.

Lewellyn, L., Cetera, M. and Horne-Badovinac, S. (2013) “Misshapen decreases integrin levels to promote epithelial motility and planar polarity in *Drosophila*,” *Journal of Cell Biology*, 200(6), pp. 721–729. Available at: https://doi.org/10.1083/jcb.201209129.

Li, Y. *et al.* (2012) “Mechanisms of Regulating Cell Topology in Proliferating Epithelia: Impact of Division Plane, Mechanical Forces, and Cell Memory,” *PLOS ONE*, 7(8), p. e43108. Available at: https://doi.org/10.1371/journal.pone.0043108.

Lynch, H.E. *et al.* (2013) “Cellular mechanics of germ band retraction in *Drosophila*,” *Developmental Biology*, 384(2), pp. 205–213. Available at: https://doi.org/10.1016/j.ydbio.2013.10.005.

Mao, Y. *et al.* (2013) “Differential proliferation rates generate patterns of mechanical tension that orient tissue growth,” *The EMBO Journal*, 32(21), pp. 2790–2803. Available at: https://doi.org/10.1038/emboj.2013.197.

Marcinkevicius, E. and Zallen, J.A. (2013) "Regulation of cytoskeletal organization and junctional remodeling by the atypical cadherin Fat," *Development*, 140(2), pp. 433–443. Available at: https://doi.org/10.1242/dev.083949.

Márquez, E.J. *et al.* (2012) "The Measurement of Local Variation in Shape," *Evolutionary Biology*, 39(3), pp. 419–439. Available at: https://doi.org/10.1007/s11692-012-9159-6.

Mashburn, D.N. *et al.* (2012) "Enabling user-guided segmentation and tracking of surface-labeled cells in time-lapse image sets of living tissues," *Cytometry Part A*, 81A(5), pp. 409–418. Available at: https://doi.org/10.1002/cyto.a.22034.

Matakatsu, H. and Blair, S.S. (2012) "Separating planar cell polarity and Hippo pathway activities of the protocadherins Fat and Dachsous," *Development*, 139(8), pp. 1498–1508. Available at: https://doi.org/10.1242/dev.070367.

Matis, M., Axelrod, J.D. and Galic, M. (2012) "A universal analysis tool for the detection of asymmetric signal distribution in microscopic images," *Developmental Dynamics*, 241(8), pp. 1301–1309. Available at: https://doi.org/10.1002/dvdy.23818.

Mirkovic, I., Pylawka, S. and Hudspeth, A.J. (2012) "Rearrangements between differentiating hair cells coordinate planar polarity and the establishment of mirror symmetry in lateral-line neuromasts," *Biology Open*, 1(5), pp. 498–505. Available at: https://doi.org/10.1242/bio.2012570.

Morishita, Y. and Hironaka, K. (2013) "Systems approach to developmental biology – designs for robust patterning," *IET Systems Biology*, 7(2), pp. 38–49. Available at: https://doi.org/10.1049/iet-syb.2012.0042.

Mouri, K., Horiuchi, S. and Uemura, T. (2012) "Cohesin controls planar cell polarity by regulating the level of the seven-pass transmembrane cadherin Flamingo," *Genes to Cells*, 17(6), pp. 509–524. Available at: https://doi.org/10.1111/j.1365-2443.2012.01604.x.

Noël, E.S. *et al.* (2013) "A Nodal-independent and tissue-intrinsic mechanism controls heart-looping chirality," *Nature Communications*, 4(1), p. 2754. Available at: https://doi.org/10.1038/ncomms3754.

Olguín, P., Glavic, A. and Mlodzik, M. (2011) "Intertissue Mechanical Stress Affects Frizzled-Mediated Planar Cell Polarity in the Drosophila Notum Epidermis," *Current Biology*, 21(3), pp. 236–242. Available at: https://doi.org/10.1016/j.cub.2011.01.001.

Pan, G. *et al.* (2013) “Signal transduction by the Fat cytoplasmic domain,” *Development*, 140(4), pp. 831–842. Available at: https://doi.org/10.1242/dev.088534.

Panousopoulou, E. *et al.* (2013) “The distribution of Dishevelled in convergently extending mesoderm,” *Developmental Biology*, 382(2), pp. 496–503. Available at: https://doi.org/10.1016/j.ydbio.2013.07.012.

Petrov, V. and Aegerter, C. (2011) “INTEGRATING MECHANICAL CONTROL THEORY INTO MODELS OF BIOLOGICAL DEVELOPMENT — ANALYTICAL REVIEW,” *Journal of Mechanics in Medicine and Biology*, 11(04), pp. 713–734. Available at: https://doi.org/10.1142/S0219519411004162.

Rolfe, R., Roddy, K. and Murphy, P. (2013) “Mechanical Regulation of Skeletal Development,” *Current Osteoporosis Reports*, 11(2), pp. 107–116. Available at: https://doi.org/10.1007/s11914-013-0137-4.

Sagner, A. *et al.* (2012) “Establishment of Global Patterns of Planar Polarity during Growth of the Drosophila Wing Epithelium,” *Current Biology*, 22(14), pp. 1296–1301. Available at: https://doi.org/10.1016/j.cub.2012.04.066.

Salbreux, G. *et al.* (2012) “Coupling Mechanical Deformations and Planar Cell Polarity to Create Regular Patterns in the Zebrafish Retina,” *PLOS Computational Biology*, 8(8), p. e1002618. Available at: https://doi.org/10.1371/journal.pcbi.1002618.

Sharma, P. and McNeill, H. (2013) “Regulation of long-range planar cell polarity by Fat-Dachsous signaling,” *Development*, 140(18), pp. 3869–3881. Available at: https://doi.org/10.1242/dev.094730.

Shwartz, Y. *et al.* (2012) “Muscle contraction controls skeletal morphogenesis through regulation of chondrocyte convergent extension,” *Developmental Biology*, 370(1), pp. 154–163. Available at: https://doi.org/10.1016/j.ydbio.2012.07.026.

Sienknecht, U.J. *et al.* (2011) “Non-cell-autonomous planar cell polarity propagation in the auditory sensory epithelium of vertebrates,” *Developmental Biology*, 352(1), pp. 27–39. Available at: https://doi.org/10.1016/j.ydbio.2011.01.009.

Staple, D.B. *et al.* (2010) “Mechanics and remodelling of cell packings in epithelia,” *The European Physical Journal E*, 33(2), pp. 117–127. Available at: https://doi.org/10.1140/epje/i2010-10677-0.

Strutt, H., Warrington, S.J. and Strutt, D. (2011) “Dynamics of Core Planar Polarity Protein Turnover and Stable Assembly into Discrete Membrane Subdomains,” *Developmental Cell*, 20(4), pp. 511–525. Available at: https://doi.org/10.1016/j.devcel.2011.03.018.

Sugimura, K. *et al.* (2013) “Robustness of force and stress inference in an epithelial tissue,” *2013 35th Annual International Conference of the IEEE Engineering in Medicine and Biology Society (EMBC)*. *2013 35th Annual International Conference of the IEEE Engineering in Medicine and Biology Society (EMBC)*, Osaka: IEEE, pp. 2712–2715. Available at: https://doi.org/10.1109/EMBC.2013.6610100.

Sugimura, K. and Ishihara, S. (2013) “The mechanical anisotropy in a tissue promotes ordering in hexagonal cell packing,” *Development*, 140(19), pp. 4091–4101. Available at: https://doi.org/10.1242/dev.094060.

Vasilica Stirbat, T. *et al.* (2013) “Multicellular aggregates: a model system for tissue rheology,” *The European Physical Journal E*, 36(8), p. 84. Available at: https://doi.org/10.1140/epje/i2013-13084-1.

Viktorinová, I. *et al.* (2011) “Modelling planar polarity of epithelia: the role of signal relay in collective cell polarization,” *Journal of The Royal Society Interface*, 8(60), pp. 1059–1063. Available at: https://doi.org/10.1098/rsif.2011.0117.

Warrington, S.J., Strutt, H. and Strutt, D. (2013) “The Frizzled-dependent planar polarity pathway locally promotes E-cadherin turnover via recruitment of RhoGEF2,” *Development*, 140(5), pp. 1045–1054. Available at: https://doi.org/10.1242/dev.088724.

Wibowo, I. *et al.* (2011) “Compartmentalized Notch signaling sustains epithelial mirror symmetry,” *Development*, 138(6), pp. 1143–1152. Available at: https://doi.org/10.1242/dev.060566.

Wu, J. *et al.* (2013) “Wg and Wnt4 provide long-range directional input to planar cell polarity orientation in Drosophila,” *Nature Cell Biology*, 15(9), pp. 1045–1055. Available at: https://doi.org/10.1038/ncb2806.

Zhu, H. and Owen, M.R. (2013) “Damped propagation of cell polarization explains distinct PCP phenotypes of epithelial patterning,” *Scientific Reports*, 3(1), p. 2528. Available at: https://doi.org/10.1038/srep02528.

Žigman, M. *et al.* (2011) “Zebrafish Neural Tube Morphogenesis Requires Scribble-Dependent Oriented Cell Divisions,” *Current Biology*, 21(1), pp. 79–86. Available at: https://doi.org/10.1016/j.cub.2010.12.005.
